# Nme protein family evolutionary history, a vertebrate perspective

**DOI:** 10.1186/1471-2148-9-256

**Published:** 2009-10-23

**Authors:** Thomas Desvignes, Pierre Pontarotti, Christian Fauvel, Julien Bobe

**Affiliations:** 1 INRA, UR1037 SCRIBE, IFR140, Ouest-Genopole, F-35000 Rennes, France; 2 IFREMER, LALR, F-34250 Palavas Les Flots, France; 3 UMR 6632/IFR48 Université de Aix Marseille/CNRS. Equipe Evolution biologique et Modélisation, case 19, 3 place Victor Hugo, 13331 Marseille Cedex 03, France

## Abstract

**Background:**

The Nme family, previously known as Nm23 or NDPK, is involved in various molecular processes including tumor metastasis and some members of the family, but not all, exhibit a Nucleoside Diphosphate Kinase (NDPK) activity. Ten genes are known in humans, in which some members have been extensively studied. In non-mammalian species, the Nme protein family has received, in contrast, far less attention. The picture of the vertebrate Nme family remains thus incomplete and orthology relationships with mammalian counterparts were only partially characterized. The present study therefore aimed at characterizing the Nme gene repertoire in vertebrates with special interest for teleosts, and providing a comprehensive overview of the Nme gene family evolutionary history in vertebrates.

**Results:**

In the present study, we present the evolutionary history of the Nme family in vertebrates and characterize the gene family repertoire for the first time in several non-mammalian species. Our observations show that vertebrate *Nme *genes can be separated in two evolutionary distinct groups. *Nme1*, *Nme2*, *Nme3*, and *Nme4 *belong to Group I while vertebrate *Nme5*, *Nme6*, *Nme7*, *Nme8*, and *Nme9 *belong to Group II. The position of *Nme10 *is in contrast more debatable due to its very specific evolutionary history. The present study clearly indicates that *Nme5*, *Nme6*, *Nme7*, and *Nme8 *originate from duplication events that occurred before the chordate radiation. In contrast, *Nme *genes of the Group I have a very different evolutionary history as our results suggest that they all arise from a common gene present in the chordate ancestor. In addition, expression patterns of all zebrafish *nme *transcripts were studied in a broad range of tissues by quantitative PCR and discussed in the light of the function of their mammalian counterparts.

**Conclusion:**

This work offers an evolutionary framework that will pave the way for future studies on vertebrate Nme proteins and provides a unified vertebrate Nme nomenclature that is consistent with the nomenclature in use in mammals. Based on protein structure and expression data, we also provide new insight into molecular functions of Nme proteins among vertebrates and raise intriguing questions on the roles of Nme proteins in gonads.

## Background

The first descriptions of Nucleoside Diphosphate Kinase (NDPK) activity, that corresponds to the phosphoryl transfer from a nucleoside triphosphate to a nucleoside diphosphate, were made in pigeon breast muscle [1] and yeast [2]. Sequences encoding for proteins with putative [3] or experimentally validated [4-6] NDPK activity were subsequently identified. These proteins, originally named NDPK based on their NDPK activity, belong to the Nme protein family according to current official gene nomenclature [7-10]. These proteins "expressed in non-metastatic cell", and thus named Nme, were also previously known as Nm23 proteins. In humans, the NME family is composed of ten genes and some of the proteins, but not all, exhibit NDPK activity.

*Nme *genes were first identified in mouse [11] and in the fruit fly *Drosophila melanogaster *[12] in which they drew attention for their surprising implication in tumor metastasis process [11] and in normal fly development [12] respectively. Soon, several orthologs of these genes were identified in other organisms ranging from the bacteria *Escherichia coli *[13] to humans [14]. They were subsequently studied for their role as tumor metastasis suppressor or enhancer depending on the cancer type. To date, ten genes displaying partial or complete NDPK domains have been identified in humans (reviewed in [15]). Proteins of this family were classified into two groups based on sequence characteristics and NDPK activity [15]. Group I Nme proteins (Nme1 to 4) display a particularly well conserved domain and active site, whereas Group II Nme proteins (Nme5 to 10) display highly divergent domains and all of them, except Nme6, lack NDPK activity [15]. In fish and amphibians, proteins of the Nme family have been implicated in key developmental processes in the oocyte or embryo [16-18]. However, the Nme proteins repertoire remains uncharacterized in almost all non-mammalian vertebrates. In teleost fish, only two Nme sequences were reported [18,19]. In non-mammalian species, the picture of the Nme family remains fuzzy and the orthology relationships of reported Nme proteins with their mammalian counterparts were only partially characterized [18,20]. Therefore, the evolutionary process which gave rise to such a complex gene family remains poorly understood and requires a complete characterization that will pave the way for future investigations of the roles of Nme proteins in vertebrates.

In the present study, we describe the evolutionary history of the *Nme *gene family in chordates and provide, for the first time, a comprehensive characterization of the *Nme *gene repertoire in vertebrates.

## Results and Discussion

### Evolutionary history of *Nme *gene family in vertebrates

Nucleoside disphosphate (NDP) kinase activity is ubiquitously found in organisms from bacteria to humans. In humans, ten *NME *genes exist that have been separated in two groups based on their amino-acid sequence [15]. These two groups originate from a gene duplication of a single NDPK ancestor gene that probably occurred before or around the metazoan radiation [21]. As indicated above, the evolutionary history of vertebrate Nme proteins has received very little attention as most existing studies focused on mammalian proteins or on specific members of the family [15,18,20,21]. Some information is however available in cellular slime molds [22], drosophila and *C. elegans *[21]. In contrast, available data in chordates and non-mammalian vertebrate species are extremely limited apart from the report of several Nme sequences [18-20].

#### A two group classification

**Figure 1:**
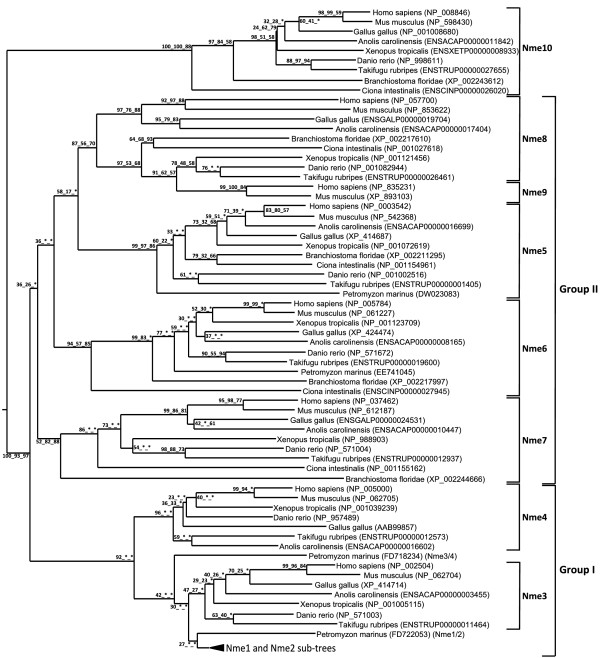
**Phylogenetic reconstruction of the Nme protein family in chordates**. The phylogenetic tree was constructed from a single multiple alignment. Bootstrap values for neighbor joining, maximum parsimony, and maximum likelihood methods, respectively, are indicated for each node. * indicates that the node does not exist in the corresponding tree. The consensus tree was calculated with the FIGENIX [87] automated phylogenomic annotation pipeline. Nme1-Nme2 subtree was removed from the main tree and studied separately (see Figure 5) for tetrapods and teleosts because of high sequence similarity and different evolutionary history. For each sequence, NCBI or Ensembl accession number and species are shown.

**Figure 2:**
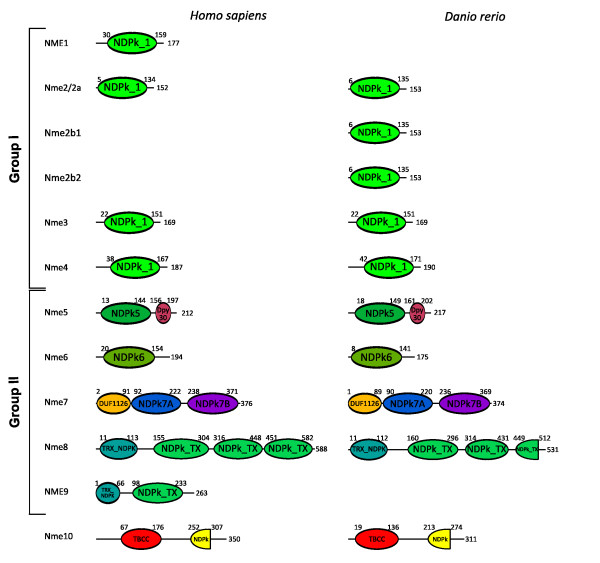
**Proteins domains of human and zebrafish Nme proteins**. The Genbank Conserved Domain Database was used for protein domain characterization [23]. Amino acid numbers corresponding to the beginning and the end of each domain are indicated and the total amino acid length number of the protein is shown at the end of each protein.

**Figure 3:**
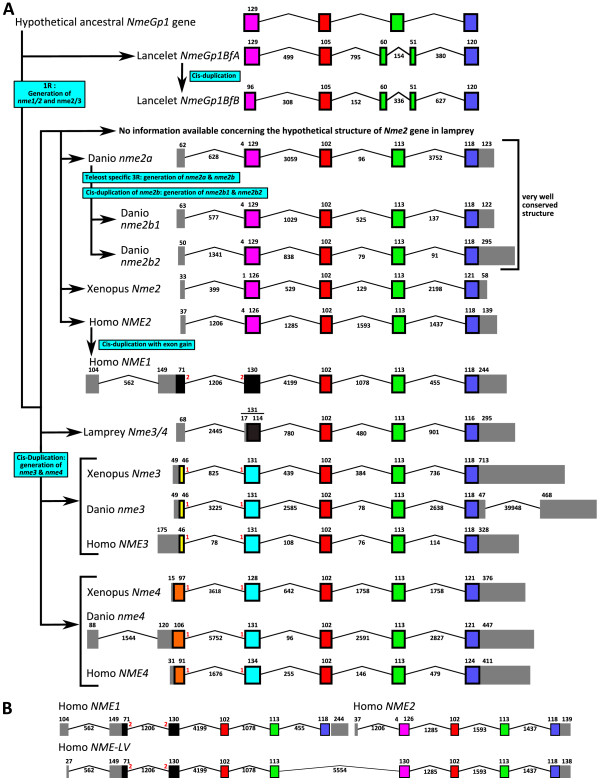
**Intron-Exon structure of Group I *Nme *genes in chordates**. (A) Intron-Exon structure and evolutionary depiction of Group I *Nme *gene structure among chordate lineage. (B) Intron-Exon structure of human *NMELV *transcript compared to Intron-Exon structure of human *NME1 *and *NME2*. Intron-exon structure was obtained using Ensembl database, or, when no information was available, by Blat of protein and cDNA sequences on genomes assemblies. Boxes correspond to exons. Non-coding exons are shown in grey. The size of introns and exons in nucleotides is shown. Introns are not drawn to scale.

**Figure 4:**
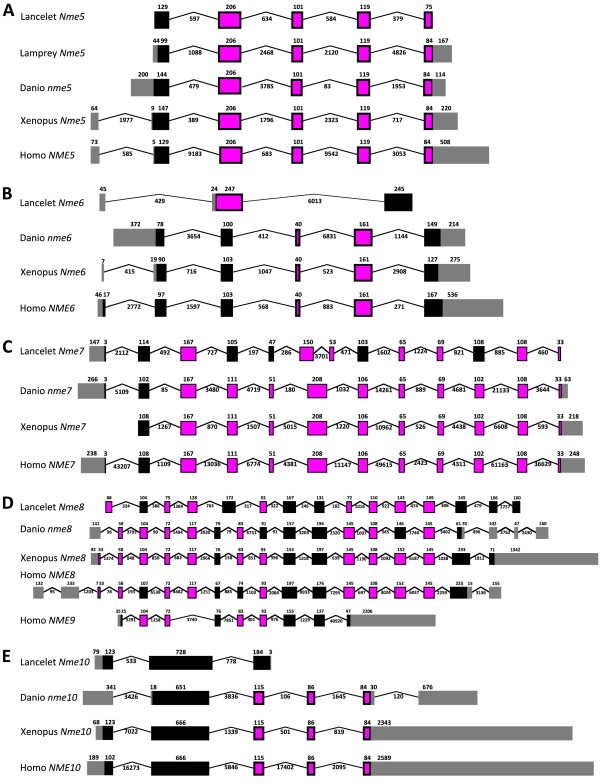
**Intron-Exon structure of Group II *Nme *genes in chordates**. Intron-Exon structure of *Nme5 *(A), *Nme6 *(B), *Nme7 *(C), *Nme8/NME9 *(D), and *Nme10 *(E) genes structure among chordates. Intron-exon structure was obtained using Ensembl database, or, when no information was available, by Blat of protein and cDNA sequences on genomes assemblies. Boxes correspond to exons. Non-coding exons are shown in grey. The size of introns and exons in nucleotides is shown. Introns are not drawn to scale.

The phylogenetic analysis of Nme proteins (Fig. 1) shows two strongly supported distinct clusters. Nme1, Nme2, Nme3, and Nme4 belong to the Group I cluster while Nme5, Nme6, Nme7, Nme8 and Nme9 belong to the Group II cluster. Within each group, all Nme subtypes are also distinctly separated from each other, with the exception of Nme9 sequences that are only found in eutherians and appear to be closely related to Nme8 sequences (Fig. 1). The analysis of the domain structure of Nme proteins using the NCBI Conserved Domain Database [23] clearly demonstrates the existence of two distinct groups among Nme1 to 9 proteins (Fig. 2) that clearly possess distinct domains. Proteins of the Group I (Nme1 to 4, Table 1) display a single type NDPk_1 domain while proteins of the Group II (Nme5 to 9, Table 2) display a single or several NDPk domains of different types, associated or not with extra-domains. For all Nme, the sequence structure, including domain(s) nature(s), length or position in the sequence, as well as the exon-intron structure (Fig. 3A &4), is highly conserved between human and zebrafish (*Danio rerio*) proteins. Together, our results on exon-intron structure, protein domains, and phylogenetic analysis, clearly indicate that the separation of vertebrate Nme1 to Nme9 proteins in two groups that has been proposed in mammals [15] is also valid for all vertebrates.

**Table 1:** Group I Nme proteins: names and symbols by species, accession numbers and corresponding chromosomal location

	**Species**	**Name**	**Other names**	**GenBank Acc #**	**Ensembl Acc #**	**Localisation**	**Position**
	***C. intestinalis***	NmeGp1CiA	NDK B	XP_002123476	ENSCINP00000011619	Chr 8q	5,908,347-5,908,808
	***C. intestinalis***	NmeGp1CiB		XP_002121438	ENSCINP00000002194	Chr 2q	7,888,026-7,888,562
***NmeGp1***	***B. floridae***	NmeGp1BfA		XP_002206993		Chr Un	116,102,546-116,104,838
	***B. floridae***	NmeGp1BfB		XP_002206992		Chr Un	116,092,575-116,094,429
	***P. marinus***	Nme2		FD722053*			
	***P. marinus***	Nme3/4		FD718234*		Contig24671	6,345-8,952

	***H. sapiens***	NME1	NDK A; NM23-H1; GAAD	NP_937818	ENSP00000337060	Chr 17	46,585,919-46,594,449
	***M. musculus***	Nme1	NDK A; Nm23-M1	NP_032730	ENSMUSP00000021220	Chr 11	93,820,547-93,829,574
	***B. taurus***	Nme1	NDK A; NDKA2; NBR-A	NP_991387	ENSBTAP00000006104	Chr 19	36,634,791-36,645,841
***Nme1***	***M. domestica***	Nme1	LOC100012868	XP_001363771	ENSMODP00000015750	Chr 2	184,090,725-184,104,175
	***O. anatinus***	Nme1			ENSOANP00000018628	SuperContig 18222	1,054-2,769
	***G. gallus***	Nme1	Nm23A	XP_420097	ENSGALP00000011811	Chr18	9,930,932-9,933,309
	***A. carolinensis***	Nme1			ENSACAESTP00000008767	Scaffold_268	1,710,978-1,717,896

	***H. sapiens***	NME2	NDK B; NM23-H2; PUF	NP_001018149	ENSP00000376888	Chr 17	46,598,821-46,604,103
	***M. musculus***	Nme2	NDK B; Nm23-M2	NP_032731	ENSMUSP00000021217	Chr 11	93,811,130-93,817,195
	***B. taurus***	Nme2	NDK B; PUF	NP_001069844		Chr 19	36,625,295-36,629,092
***Nme2***	***M. domestica***	Nme2	NDK B	XP_001363684	ENSMODP00000015743^†^	Chr 2	184,076,307-184,081,978
	***O. anatinus***	Nme2			ENSOANP00000018629	SuperContig 18222	6,818-8,864
	***G. gallus***	Nme2	CNDPK; NDK_CHICK	NP_990378	ENSGALP00000034078	Chr18	5,062,096-5,064,054
	***A. carolinensis***	Nme2			ENSACAESTP00000008779	Scaffold_268	1,724,830-1,729,637
	***X. tropicalis***	Nme2	NME1	NP_001005140	ENSXETP00000024764	Scaffold_673	77,640-81,451

	***H. sapiens***	NMELV	NME1-NME2	NP_001018146	ENSP00000376894	Chr 17	49,230,997-49,249,103
	***P. troglodytes***	NmeLV	NME1-NME2	XP_511889	ENSPTRP00000044657	Chr 17	50,152,238-50,171,443
***Nme-LV***	***E. caballus***	NmeLV	NME1-NME2	XP_001499951	ENSECAP00000019175^†^	Chr 11	26,692,478-26,707,469
	***B. taurus***	NmeLV	NDKB-BOVIN		ENSBTAP00000041066	Chr 19	36,625,292-36,645,867
	***O. anatinus***	NmeLV	NME1-NME2	XP_001515701		SuperContig18222	1057-8751
	***A. carolinensis***	NmeLV			ENSACAP00000002161	Scaffold_268	1,706,438-1,729,637

	***D. rerio***	Nme2a	NM23B; nme2l	NP_956264	ENSDARP00000064338	Chr 20	5,310,868-5,319,057
	***O. latipes***	Nme2a			ENSORLP00000018429	Chr 19	21,821,808-21,827,808
***Nme2a***	***G. aculeatus***	Nme2a			ENSGACP00000020117	Scaffold_48	706,609-708,477
	***T. nigroviridis***	Nme2a		CAF90396^†^	ENSTNIP00000005730	Chr Un_random	34,843,841-34,848,064
	***T. rubripes***	Nme2a			ENSTRUP00000014520	Scaffold_29	678,695-680,960

	***D. rerio***	Nme2b1	nme2; nme1; ndpkz1; NM23B	NP_571001	ENSDARP00000074169^†^	Scaffold Zv7_NA1913	1,772-2,772
***Nme2b***	***D. rerio***	Nme2b2	Ndpkz2	NP_571002		Scaffold Zv7_NA1913	8,750-9,346
	***O. latipes***	Nme2b	NDKA; GAAD; NME1-NME2		ENSORLP00000023099	Scaffold1014	323-3,121
	***T. rubripes***	Nme2b	NDKA; GAAD		ENSTRUP00000001870	Scaffold_307	158,498-160,002

	***H. sapiens***	NME3	NDPKC; DR-nm23; NM23-H3	NP_002504	ENSP00000219302	Chr 16	1,760,323-1,761,711
	***M. musculus***	Nme3	NDPKC; DR-nm23; Nm23-M3	NP_062704	ENSMUSP00000024978	Chr 17	25,033,459-25,034,448
	***G. gallus***	Nme3	NDPKC; DR-nm23	XP_414714	ENSGALP00000003531	Chr 14	13,989,877-13,991,647
	***A. carolinensis***	Nme3	NDPKC; NDK 3; DR-nm23		ENSACAP00000003455	Scaffold_1065	123,674-128,641
***Nme3***	***X. tropicalis***	Nme3	MGC89980	NP_001005115	ENSXETP00000022770	Scaffold_27	933,363-937,018
	***D. rerio***	Nme3	ndpkz3; NDPK-Z3	NP_571003	ENSDARP00000075112	Chr 3	13,184,712-13,194,308
	***O. latipes***	Nme3	NDK 3; NDPKC; DR-nm23		ENSORLP00000014699	Chr 8	15,106,871-15,109,573
	***G. aculeatus***	Nme3	NDK 3; NDPKC; DR-nm23		ENSGACP00000017241	GroupXI	12,674,486-12,677,154
	***T. rubripes***	Nme3	NDK 3; NDPKC; DR-nm23		ENSTRUP00000011464	Scaffold_294	107,469-108,977
	***T. nigroviridis***	Nme3	NDK 3; NDPKC; DR-nm23	CAG02649	ENSTNIP00000014528	Chr Un_random	6,812,965-6,814,848

	***H. sapiens***	NME4	NDPKD; NM23-H4	NP_005000	ENSP00000219479	Chr 16	387,193-390,754
	***M. musculus***	Nme4	NDPKD; Nm23-M4	NP_062705	ENSMUSP00000025007	Chr 17	26,228,682-26,232,433
	***G. gallus***	Nme4	NDPKD	AAB99857		Chr 14	13,763,108-13,764,673
	***A. carolinensis***	Nme4			ENSACAP00000016602	scaffold_1361	31,704-32,514
***Nme4***	***X. tropicalis***	Nme4		NP_001039239	ENSXETP00000022726	Scaffold_27	1,305,654-1,312,714
	***D. rerio***	Nme4	zgc:56482	NP_957489	ENSDARP00000060403	Chr 3	14,178,447-14,192,697
	***O. latipes***	Nme4	NDPKD		ENSORLP00000014089	Chr 8	14,902,635-14,905,718
	***G. aculeatus***	Nme4	NDPKD		ENSGACP00000016999	GroupXI	12,533,938-12,535,079
	***T. rubripes***	Nme4	NDPKD		ENSTRUP00000012573	Scaffold_112	81,060-82,895
	***T. nigroviridis***	Nme4	NDPKD	CAG12673	ENSTNIP00000022136	Chr Un_random	7,826,243-7,828,703

Protein names were retrieved from Genbank, Ensembl, iHOP and ZFIN. Location was obtained using Ensembl genome browser, or by UCSC Genome Bioinformatics BLAT when not available on Ensembl. *, sequence is an EST. †, incomplete sequence.

**Table 2:** Group II Nme proteins: names and symbols by species, accession numbers and corresponding chromosomal location

	**Species**	**Name**	**Other names**	**GenBank Acc #**	**Ensembl Acc #**	**Localisation**	**Position**
	***C. intestinalis***	Nme5	ci-ndk/dpy26	NP_001154961	ENSCINP00000008954^†^	Chr 7q	1,031,987-1,036,756
	***B. floridae***	Nme5		XP_002211295		Chr Un	731,166,868-731,169,160
	***P. marinus***	Nme5		DW023083*		Contig18268	2,311-13,400
	***H. sapiens***	NME5	NDK-H5; NM23-H5; IPIA-β	NP_003542	ENSP00000265191	Chr 5	137,478,761-137,503,031
	***M. musculus***	Nme5	NDK-M5; Nm23-M5	NP_542368	ENSMUSP00000078269	Chr 18	34,722,295-34,738,760
***Nme5***	***G. gallus***	Nme5	NDP kinase homolog 5; IPIA-β	XP_414687	ENSGALP00000022919	Chr 13	14,517,448-14,548,478
	***A. carolinensis***	Nme5	NDP kinase homolog 5; IPIA-β		ENSACAP00000016699	Scaffold_29	3,794,500-3,804,710
	***X. tropicalis***	Nme5	NDPK homolog 5; IPIA-β	NP_001072619	ENSXETP00000008322	Scaffold_65	2,613-8,494
	***D. rerio***	Nme5	zgc:92812	NP_001002516	ENSDARP00000060997	Chr 14	55,814,542-55,821,523
	***O. latipes***	Nme5	NDP kinase homolog 5; IPIA-β		ENSORLP00000006672	Chr 10	13,043,372-13,046,033
	***G. aculeatus***	Nme5	NDP kinase homolog 5; IPIA-β		ENSGACP00000023932	GroupIV	11,477,537-11,479,481
	***T. rubripes***	Nme5	NDP kinase homolog 5; IPIA-β		ENSTRUP00000001405	Scaffold_126	261,197-263,042
	***T. nigroviridis***	Nme5	NDP kinase homolog 5; IPIA-β	CAG01205	ENSTNIP00000013421	Chr 1	943,305-945,715

	***C. intestinalis***	Nme6	NDK 6; IPIA-α	XP_002129729	ENSCINP00000027945^†^	Scaffold_1779	5,509-6,021
	***B. floridae***	Nme6		XP_002217997		Chr Un	307,935,668-307,942,172
	***P. marinus***	Nme6		EE741045*			
	***H. sapiens***	NME6	IPIA-α; NDK-H6; NM23-H6	NP_005784	ENSP00000307125	Chr 3	48,310,595-48,317,852
	***M. musculus***	Nme6	NDK-6; Nm23-M6	NP_061227	ENSMUSP00000035053	Chr 9	109,735,308-109,745,475
***Nme6***	***G. gallus***	Nme6	NDK 6; IPIA-α	XP_424474	ENSGALP00000015875	Chr 1	96,907-98,947
	***A. carolinensis***	Nme6	NDK 6; IPIA-α		ENSACAP00000008165	Scaffold_2735	3,138-10,113
	***X. tropicalis***	Nme6	NDK 6; IPIA-α	NP_001123709	ENSXETP00000034257	Scaffold_857	287,305-293,502
	***D. rerio***	Nme6	Ndpkz6	NP_571672	ENSDARP00000094574	Chr 20	19,668,343-19,681,489
	***O. latipes***	Nme6	NDK 6; IPIA-α		ENSORLP00000020366	Chr 24	13,630,759-13,633,495
	***G. aculeatus***	Nme6	NDK 6; IPIA-α		ENSGACP00000014120	GroupXVIII	10,683,098-10,684,634
	***T. rubripes***	Nme6	NDK 6; IPIA-α		ENSTRUP00000019600	Scaffold_72	321,734-323,012
	***T. nigroviridis***	Nme6	NDK 6; IPIA-α	CAG09120^†^	ENSTNIP00000019439	Chr 14	5,693,013-5,693,908

	***C. intestinalis***	Nme7	NDK/DM44	NP_001155162	ENSCINP00000025129^†^	Chr 1p	3,423,461-3,424,231
	***B. floridae***	Nme7		XP_002244666		Chr Un	788,282,087-788,292,883
	***H. sapiens***	NME7	NDK-7; NM23-H7	NP_037462	ENSP00000356785	Chr 1	167,368,399-167,603,810
	***M. musculus***	Nme7	NDK-7; Nm23-M7	NP_612187	ENSMUSP00000027862	Chr 1	166,237,803-166,334,805
	***G. gallus***	Nme7	NDK-7		ENSGALP00000024531	Chr 1	87,015,645-87,088,484
***Nme7***	***A. carolinensis***	Nme7	NDK-7		ENSACAP00000008165	Scaffold_2735	3,138-10,113
	***X. tropicalis***	Nme7	MGC75677	NP_988903	ENSXETP00000005150	Scaffold_169	1,646,165-1,680,298
	***D. rerio***	Nme7	Ndpkz4; Ndpkz7	NP_571004	ENSDARP00000073091	Chr 6	20,659,126-20,718,810
	***O. latipes***	Nme7		DK039970*	ENSORLP00000019661^†^	Chr 4	29,680,976-29,697,346
	***G. aculeatus***	Nme7	NDK 7		ENSGACP00000018067	GroupVIII	17,708,704-17,717,807
	***T. rubripes***	Nme7	NDK 7		ENSTRUP00000012937	Scaffold_13	352,791-361,358
	***T. nigroviridis***	Nme7	NDK 7		ENSTNIP00000004782	Chr 1	10,762,024-10,774,089

	***C. intestinalis***	Nme8	TXNDC3; CiIC3	NP_001027618	ENSCINP00000013583	Chr 9q	3,694,058-3,703,935
	***B. floridae***	Nme8		XP_002217610		Chr Un	302,074,149-302,085,811
	***H. sapiens***	NME8	TXNDC3; CILD6; NM23-H8; SPTRX2	NP_057700	ENSP00000199447	Chr 7	37,854,724-37,906,525
	***M. musculus***	Nme8	Txndc3; Sptrx2	NP_853622	ENSMUSP00000089358	Chr 13	19,736,950-19,789,629
	***G. gallus***	Nme8	TXNDC3	XP_426021	ENSGALP00000019704	Chr 2	46,221,676-46,240,899
***Nme8***	***A. carolinensis***	Nme8	TXNDC3; Sptrx-2		ENSACAP00000017404	Scaffold_28	5,222,555-5,265,400
	***X. tropicalis***	Nme8	TXNDC6; Txl-2	NP_001121456	ENSXETP00000002355	Scaffold_664	444,670-467,036
	***D. rerio***	Nme8	zgc:162216	NP_001082944		Chr13	17,124,355-17,152,394
	***O. latipes***	Nme8	TXNDC6; Txl-2		ENSORLP00000020619	Chr 21	22,030,361-22,038,307
	***G. aculeatus***	Nme8	TXNDC6; Txl-2		ENSGACP00000008024	GroupXVI	11,682,650-11,688,299
	***T. rubripes***	Nme8	TXNDC6; Txl-2		ENSTRUP00000026461	Scaffold_46	902,376-907,179
	***T. nigroviridis***	Nme8	TXNDC6; Txl-2	CAG09297	ENSTNIP00000019567	Chr 2	14,973,997-14,979,390

	***H. sapiens***	Nme9	TXNDC6, TXL-2	NP_835231	ENSP00000321929	Chr 3	137,980,279-138,048,205
***Nme9***	***M. musculus***	Nme9	TXL-2	XP_893103		Chr 9	99,360,108-99,371,350
	***B. taurus***	Nme9	TXNDC6	NP_001069083	ENSBTAP00000017071	Chr 1	132,828,887-132,856,623

	***C. intestinalis***	Nme10	XRP2	XP_002121234^†^	ENSCINP00000026020	Chr 14q	3,012,876-3,017,751
	***B. floridae***	Nme10		XP_002243612		Chr Un	778,883,396-778,885,823
	***H. sapiens***	NME10	RP2; TBCCD2	NP_008846	ENSP00000218340	Chr X	46,696,347-46,741,793
	***M. musculus***	Nme10	Rp2h	NP_598430	ENSMUSP00000111049	Chr X	19,941,607-19,982,781
	***G. gallus***	Nme10	RP2; XRP2_CHICK	NP_001008680	ENSGALP00000026942	Chr 1	134,314,101-134,332,289
***Nme10***	***A. carolinensis***	Nme10	XRP2		ENSACAP00000011842	Scaffold_571	329,765-343,988
	***X. tropicalis***	Nme10	XRP2		ENSXETP00000008933	Scaffold_253	956,431-969,596
	***D. rerio***	Nme10	RP2	NP_998611	ENSDARP00000065116	Chr 6	40,312,188-40,323,321
	***O. latipes***	Nme10	XRP2		ENSORLP00000020082	Chr 4	30,358,786-30,362,942
	***G. aculeatus***	Nme10	RP2		ENSGACP00000018275	GroupVIII	18,001,999-18,006,009
	***T. rubripes***	Nme10	XRP2		ENSTRUP00000027655	Scaffold_13	677,342-680,474
	***T. nigroviridis***	Nme10	XRP2	CAG01390	ENSTNIP00000013571	Chr 1	11,019,475-11,022,175

Protein names were retrieved from Genbank, Ensembl, iHOP and ZFIN. Location was obtained using Ensembl genome browser, or by UCSC Genome Bioinformatics BLAT when not available on Ensembl. *, sequence is an EST. ^†^, incomplete sequence.

#### Nme10, the outgroup of the family

Nme10 protein, previously named *X-linked Retinitis Pigmentosa 2 *(*XRP-2*), is the most recently identified member of the Nme family and vertebrate Nme10 proteins form a specific group as shown by the phylogenetic analysis (Fig. 1). It is also noteworthy that sequence identities between prochordates and vertebrates range from 34.5% to 58.2%, indicating a high divergence between prochordate and vertebrate proteins in comparison to the high sequence identity observed among vertebrates species (i.e. 60.9% to 93%) [See Additional file 1]. The protein domain analysis reveals that all vertebrate Nme10 only possess a partial NDPk domain (Fig. 2), which is not present in either Ciona (*Ciona intestinalis*) or lancelet (*Branchiostoma floridae*) Nme10 proteins (data not shown). The comparison of the exon-intron structure of the *Nme10 *gene between lancelet and vertebrates (Fig. 4E) clearly shows that the addition of the partial NDPk domain in vertebrates is associated with a different number of exons in the 3' end of the gene. Together, these observations suggest that a partial NDPk domain was inserted in the *Nme10 *gene before the gnathostome radiation. As the current status of the lamprey genome preliminary assembly did not allow us to identify any *Nme10*-related gene in lamprey we are currently unable to provide a better evaluation of the timing of the insertion of this NDPk fragment into the *Nme10 *gene in the vertebrate lineage. In summary, our observations clearly show that Nme10, in contrast to all other vertebrate Nme proteins, is characterized by a recent incorporation of an NDPk domain. However, because of the gene nomenclature used in mammals [24], we suggest to name this gene *Nme10 *in vertebrates. In contrast, the classification of this gene in the Group II is more debatable in the light of its totally different evolutionary history.

#### *Nme5*, *Nme6*, *Nme7 *and *Nme8 *originate from duplication events that occurred prior to the chordate radiation

We have been able to identify Nme5, Nme6, Nme7, and Nme8 proteins in ciona and lancelet as well as in all investigated vertebrate species, with the exception of the lamprey in which Nme7 and Nme8 could not be found in the current genome preliminary assembly. While we cannot rule out that *Nme7 *and *Nme8 *have been lost in lamprey, it is also possible that the preliminary status of the genome assembly and the relatively low sequencing coverage (5.9X) can explain why we have been unable to identify these genes. It should however be stressed that both domain (Fig. 2) and exon-intron structure (Fig. 4A-D) of *Nme5*, *Nme6*, *Nme7*, and *Nme8 *are particularly well conserved among chordates, with the exception of lancelet *Nme6 *gene that displays a very specific exon-intron structure. In addition, Nme5, Nme6, Nme7 and Nme8 proteins exhibit high degree of identity among chordates [See Additional files 1, 2, and 3]. In addition, the orthology relationships among species are also clearly supported by the phylogenetic analysis for each protein subtype (Fig 1). Together with existing data on the origin of Group II Nme proteins [21], our observations indicate that *Nme5*, *Nme6*, *Nme7*, and *Nme8 *genes originate from duplication events that occurred before the chordate radiation.

#### Nme9, a novel eutherian Nme8-related protein

The Nme9 protein was recently characterized and classified as a member of Group II [24,25]. Thus far, Nme9 has only been found in human, mouse and cow databases but not in any non-mammalian vertebrate species (Table 2). The human NME9 protein contains a Thioredoxin domain (TRX_NDPk) and an NDPk_TX domain that are also found in the N-terminus region of the human NME8 protein (Fig. 2). Similarly, *NME8 *and *NME9 *display a similar exon-intron structure in the 5'-region of the gene (Fig. 4D). It is also noteworthy that *Nme8 *and *Nme9 *genes are located on different chromosomes in both humans and mice. Based on these observations, we hypothesize that *Nme9 *originates from an incompletely translocated duplication of the *Nme8 *gene. The position of human and mouse Nme9 sequences in the phylogenetic analysis support the strong relationship between Nme9 and Nme8 (Fig. 1). The position of Nme9 sequences within the Nme8/Nme9 subtree is in contrast inconsistent with the above hypothesis. The possibility that prochordate, teleost, and amphibian Nme8 proteins would be more closely related to mammalian Nme9 proteins than to mammalian Nme8 proteins can however be ruled out by the highly conserved exon-intron structure (Fig. 4D) and domain organization (Fig. 2) of the *Nme8 *gene among chordates. Altogether, these results clearly indicate that Nme9 belongs to the Group II of the Nme proteins. Given that *Nme9 *gene could only be found in eutherians our data suggest that *Nme9 *arose from a duplication event that occurred after the separation of eutherian and metatherian groups.

#### Vertebrate Nme proteins of the Group I

In mammals, the Group I Nme is composed of Nme1, Nme2, Nme3 and Nme4 and orthologs could be identified in both anole lizard and chicken. The situation is in contrast much more complex for amphibians, teleosts, lamprey and prochordates as discussed below.

##### Gnathostome *Nme3 *and *Nme4 *originate from an *Nme3/4 *vertebrate ancestor

In *Xenopus tropicalis*, as well as in all studied teleost, orthologs of amniotes Nme3 and Nme4 proteins could be identified (Fig. 1). The phylogenetic analysis of Group I Nme proteins reveals a strongly supported divergence of Nme4 from other Nme of the Group I (Fig. 1). At the amino-acid level, Nme4 proteins exhibit sequence identities ranging from 40.2 to 85.1% among vertebrates [See Additional file 2]. Nme4 protein domain structure is also very well conserved between human and zebrafish as the domain size is equal in both species (130 aa) even though some minor differences exist in pre- and post-domain length (Fig. 2). Similarly, *Nme4 *exon-intron structure is also very well conserved in Xenopus, zebrafish and human, and differences only concern exon size in the pre-domain coding region (Fig. 3A). The phylogenetic analysis also suggests that Nme3 proteins are divergent from Nme1/Nme2 (Fig 1). Nme3 proteins display sequence identities ranging from 58.4 to 84.1% among vertebrates [See Additional file 2]. The Nme3 protein domain structure (Fig. 2) is identical in humans and zebrafish. Similarly an identical exon-intron structure (Fig. 3A) was observed in *Xenopus tropicalis*, human and zebrafish *nme3 *genes. Together, these observations strongly suggest that despite the low support values of the Nme3 branch on the phylogenetic tree (Fig. 1), orthologs of mammalian Nme3 proteins can be found in teleosts and amphibians. This conclusion is further supported by the phylogenetic analysis carried out using all available teleost Nme sequences regardless of the genome sequencing status of the species [See Additional file 4] in which high bootstrap values support the Nme3 branch.

In contrast to teleosts, amphibians and mammals, only one Nme3/Nme4-related sequence could be found in lamprey. Interestingly, the phylogenetic analysis suggests that this sequence is related to both Nme3 and Nme4 groups (Fig. 1). The exon-intron structure of this *Nme3/Nme4*-related lamprey gene reveals similarities with both *Nme3 *and *Nme4 *genes (Fig. 3A). Interestingly, when adding non-coding and coding parts, the size of the second exon of the lamprey *Nme3/Nme4*-related gene is exactly the same as the size of the second exon of Xenopus *Nme3*, zebrafish *Nme3*, human *Nme3*, and zebrafish *Nme4*. It should also be noted that for both *Nme3 *and *Nme4*, the first intron is inserted after the first base of a codon. Finally, it is noteworthy that *Nme3 *and *Nme4 *genes are always located on the same chromosome (Table 1) at very close locations in mammals, chicken, Xenopus and teleosts. Altogether, these observations suggest that, in the vertebrate ancestor, for whom the lamprey is the most closely related descendant, only one *Nme3/Nme4*-related gene existed. We hypothesize that this ancestor *Nme3/Nme4 *gene gained a start codon in the first exon after the separation of cyclostomes and gnathostomes lineages. *Nme3 *and *Nme4 *subsequently arose from a cis-duplication of this gene that occurred before or around teleost radiation. The *Nme3/Nme4*-related gene found in lamprey was thus named *Nme3/4 *to reflect its phylogenetic relationship with *Nme3 *and *Nme4 *genes.

###### An amniote specific cis-duplication of *Nme1/2 *ancestor gene

**Figure 5:**
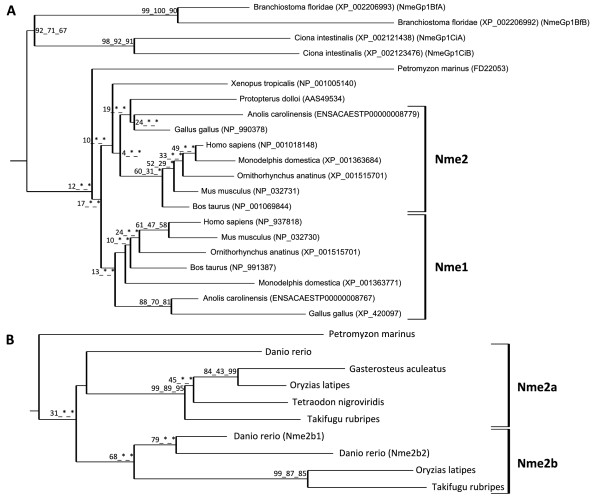
**Phylogenetic analysis of Nme1 and Nme2 proteins**. Tetrapods Nme1 and Nme2 (A) and teleost Nme2 (B) phylogenetic trees were constructed from separate multiple alignments. Bootstrap values for neighbor-joining, maximum parsimony, and maximum likelihood methods, respectively, are indicated for each node. * indicates that the node does not exist in the corresponding tree. The consensus tree was calculated with the FIGENIX automated phylogenomic annotation pipeline [87]. For each sequence, accession number and species name are shown.

**Figure 6:**
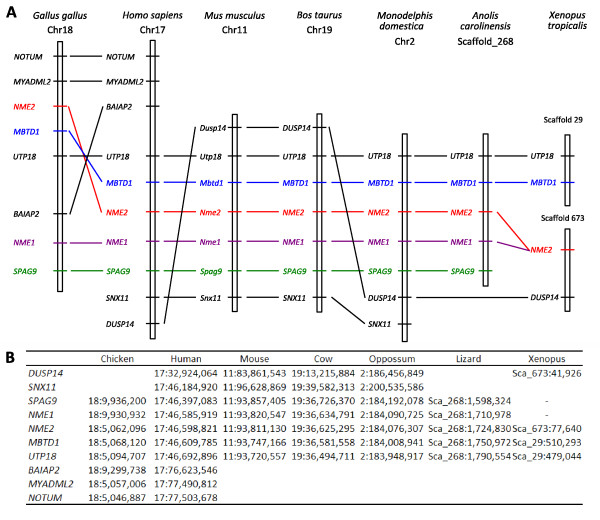
**Conserved synteny around *Nme1 *and *Nme2 *loci in tetrapods**. The syntenic relationships of genes in the vicinity of human *NME1 *and *NME2 *linked genes were established using CASSIOPE. For clarity reasons, only selected genes are shown. (A) Graphical view of syntenic relationships in chicken, human, mouse, cow, opossum, and anole lizard *Nme1 *and *Nme2 *loci vicinity. Only one *Nme2 *was found in *Xenopus tropicalis *(B) Location of each marker on the corresponding numbered chromosome or scaffold.

In contrast to Nme3 and Nme4, orthologs of both human NME1 and NME2 can only be found in amniotes and form two clusters corresponding to Nme2 and Nme1 proteins respectively (Fig. 5A). In *Xenopus tropicalis *and lungfish (*Protopterus dolloi*), only one Nme1/Nme2-related protein was identified as shown by the phylogenetic analysis. No *Nme1*-like cDNA was found among the 1.2 million *Xenopus tropicalis *ESTs available in public databases (August 2009). Within amniotes, *Nme1 *and *Nme2 *are always located on the same chromosome (Table 1). Furthermore, in mammals and lizard, *Nme1 *and *Nme2 *are always located next to each other (Fig. 6). In addition, the synteny analysis of *Nme1 *and *Nme2 *in tetrapods demonstrated that conserved genes in the vicinity of human *NME1 *and *NME2 *genes could be identified among all studied amniote species (Fig. 6). In chicken, we hypothesize that a chromosomal inversion of the chromosomic part located between *Nme1 *and *Myadl2 *resulted in the separation of the two genes. In amniotes, *Nme2 *and *Nme1 *are always linked to *Mbtd1 *and *Spag9*. In *Xenopus tropicalis*, the synteny conservation in the vicinity of *Nme2 *is less clear (Fig. 6). Nevertheless, note that *Dusp14 *is in the vicinity of *Nme2 *among all tetrapods with the exception of chicken and anole lizard. Altogether, these observations suggest that, in all studied amniote species, *Nme1 *and *Nme2 *are co-orthologs of *Xenopus tropicalis Nme1/Nme2*-related gene, and that a cis-duplication event of the ancestor gene occurred before or around amniote radiation. This observation is in total agreement with the conclusions made by Ishikawa and coworkers [21] indicating that rat and human *NME1 *and *NME2 *resulted from a cis-duplication of a common ancestor gene. This is also consistent with the previously made hypothesis of a duplication of the ancestor gene that occurred after the separation of tetrapods and fish lineages and after amphibians and amniotes divergence [18,20]. However, we cannot rule out that the cis-duplication of *Nme1/Nme2*-ancestor gene occurred before amphibian radiation. In that case, the duplication would have been followed by the loss of *Nme1 *in amphibians. However, no trace of an *Nme1 *gene could be found on *Xenopus tropicalis *genomic sequence between *Nme2 *and *Dusp4 *genes (Fig. 6). This observation would thus be in favor of the hypothesis of duplication of the *Nme1/Nme2 *ancestor gene after amphibian radiation.

####### Mammalian *Nme2 *is most closely related to the *Nme1/Nme2 *ancestor gene

Comparison of the primary structure of Nme1 and Nme2 reveals that both proteins are highly conserved among amniotes with mean amino-acid (aa) sequence identities of 83,1% and 88.5% respectively [See Additional file 5]. It is also noteworthy that Nme2 is more conserved than Nme1 among vertebrates. The phylogenetic analysis suggests that both lungfish (*Protopterus dolloi*) and Xenopus Nme1/Nme2-related proteins would be more closely related to amniote Nme2 than to Nme1 (Fig. 5A). In addition, the exon-intron structure of Xenopus *Nme1/Nme2*-related gene is highly similar to human *NME2 *exon-intron structure (Fig. 3A). This highly conserved exon-intron structure is also found in zebrafish (Fig. 3A). In contrast, human *NME1 *exon-intron structure is different from human *NME2 *and Xenopus sequences as it exhibits an additional exon at the 5' end of the gene. Together, these observations indicate that *NME2 *is most similar to the ancestor gene while *NME1 *exhibits a different exon-intron structure. For this reason, the *Xenopus tropicalis Nme1/Nme2*-related gene was named *Nme2*. This name was thus also used for *Nme1/Nme2*-related genes found in teleosts and lamprey.

######## The NmeLV form

Using the different sequence databases available in amniotes, a long variant transcript, corresponding to a read-trough transcript of *Nme1 *and *Nme2 *genes can be found in human, chimpanzee, horse, cow, platypus, and anole lizard (Table 1). In contrast, this read-through transcript could not be found in chicken in which a chromosomal inversion resulted in the separation of *Nme1 *and *Nme2 *genes on the chromosome. Interestingly, the human transcript is composed of the first four exons of *NME1 *and all *NME2 *exons (Fig. 3B). To date, the corresponding protein, Nme Long Variant (NmeLV) has only been studied in humans [26] and no information is available in other species.

######### Nme2a and Nme2b in teleosts probably emerged from 3R genome duplication and Nme2a is most similar to the vertebrate ancestor

**Figure 7:**
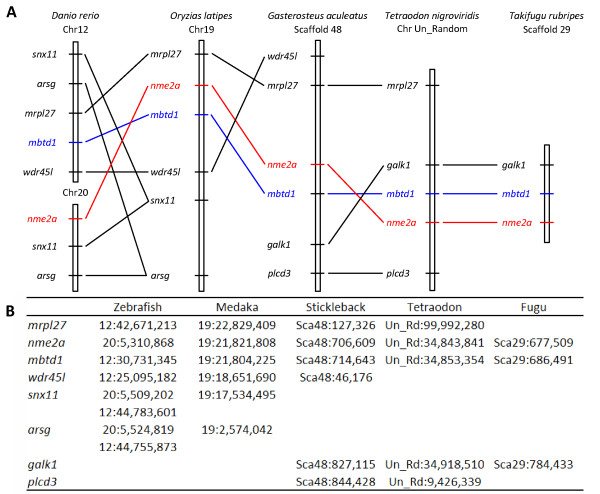
**Conserved synteny around the *nme2a *locus in teleost fish**. Syntenic relationships of genes in the vicinity of *nme2a *gene was established using Ensembl orthology informations [83]. (A) Graphical view of syntenic relationships in zebrafish, medaka, stickleback, tetraodon and fugu *nme2a *locus vicinity. (B) Location of each marker on the corresponding numbered chromosome or scaffold.

In studied teleost species, the number of *Nme1/2*-related genes varies from 1 to 3 depending on the species (Fig. 5B). As indicated above, these genes have been named *nme2 *because they are most similar to the *Nme2 *gene (Fig. 2 &3A). The phylogenetic analysis revealed that *nme2a *is present in the five teleost species with complete genome sequence, whereas *nme2b *genes could not be found in stickleback and tetraodon (Fig. 5B). In contrast, a single Nme2b protein was found in medaka (*Oryzias latipes*), and fugu (*Takifugu rubripes*) while, the phylogentic tree clearly indicates a further duplication of the *nme2b *gene in zebrafish resulting in two distinct proteins termed Nme2b1 and Nme2b2. The phylogenic analysis also suggests that Nme2a and Nme2b are co-orthologs of the lamprey Nme2. This further confirms that the lamprey *Nme2 *gene could be a direct descendant of the *Nme2 *ancestor gene (Fig. 5B). In addition, zebrafish Nme2a, Nme2b1, and Nme2b2 have exactly the same protein domain structure, with the same total length and the same NDPk_1 domain located at the same position (Fig. 2). Similarly, zebrafish *nme2a*, *nme2b1*, and *nme2b2 *have exactly the same coding exon structure (Fig. 3A). As previously indicated, the exon-intron structure is well conserved among vertebrate *Nme2 *genes and clearly distinct from the *Nme1 *gene. Conserved genes in the vicinity of *nme2a *gene in teleosts were identified among studied species by a synteny conservation study (Fig. 7). For medaka, stickleback (*Gasterosteus aculeatus*), tetraodon (*Tetraodon nigroviridis*), and fugu, the synteny is well conserved and the *mbtd1 *gene was found in the vicinity of the *nme2a *gene in agreement to what is observed in tetrapods (Fig. 7). Interestingly, Nakatani *et al *[27], demonstrated that medaka chromosome 19, on which is located *nme2a*, is orthologous to a part of human chromosome 17, on which *NME1 *and *NME2 *are located. In addition, the primary structure appears to be more conserved for Nme2a in comparison to Nme2b as they display 73.9 and 67.7% mean aa identities respectively [See Additional file 5]. Altogether, these observations suggest that among teleost *nme2 *genes, *nme2a *is most similar to the ancestor gene. In teleost, the *nme2b *gene was not found in tetraodon and stickleback, thus indicating a possible loss of this gene in both species. Furthermore, for all studied teleosts displaying *nme2a *and *nme2b*, the two paralogous genes are always located on different chromosomes or scaffolds (Table 1). Interestingly, the fugu *nme2b *gene is associated to a paralog of *mbtd1 *(data not shown), suggesting that the duplication event from which *nme2a *and *nme2b *arose in teleost is linked to the teleost-specific third round of whole genome duplication (3R). The phylogenetic analysis performed using all available Nme2 sequences in teleosts [See Additional file 6] would be in favor of this hypothesis as numerous other teleost species from different genders such as seabream (*Sparus aurata*), pike (*Esox lucius*), seabass (*Dicentrarchus labrax*), black cod (*Anoploma fimbria*), and grouper (*Epinepheles coioides*) exhibit *nme2a *and *nme2b *genes. Finally, it is noteworthy that, in contrast to *nme2*, gene duplicates resulting from 3R whole genome duplication were not retained for other teleost *nme *genes.

########## *nme2b1 *and *nme2b2 *emerged from a cis-duplication of *nme2b*

In contrast to *nme2a*, very little information is available on the position of *nme2b *genes in teleosts as they are all located on scaffolds. In zebrafish, it should nevertheless be noted that *nme2b1 *and *nme2b2 *genes are located in tandem on the same scaffold (Table 2). This suggests a cis-duplication event of zebrafish *nme2b *ancestor gene from which *nme2b1 *and *nme2b2 *genes arose.

########### The *Nme *gene repertoire in the vertebrate ancestor

**Figure 8:**
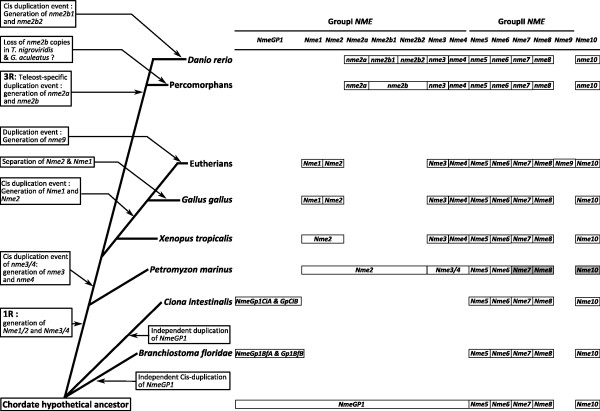
**Schematic depiction of *Nme *genes repertoire in chordates**. For each species, the repertoire of *Nme *genes is shown with the exception of lamprey in which *Nme7*, *Nme8*, and *Nme10 *could not be found in the genome preliminary assembly. These three genes were thus shaded in grey.

In order to better characterize the putative *Nme *gene repertoire of the vertebrate ancestor, we have analyzed Nme-related sequences available in the two prochordate *Ciona intestinalis *and *Branchiostoma floridae*. As discussed above, orthologs for *Nme5*, *Nme6*, *Nme7*, *Nme8 *and *Nme10 *could be identified, thus indicating that these genes emerged before chordate radiation (Fig. 1). Concerning Group I Nme, two sequences could be found in both species. In the lancelet, the genome second assembly available from the Joint Genome Institute [28], clearly shows that only two Group I *Nme *genes are present in the lancelet genome. The phylogenetic analysis (Fig. 5A), clearly indicates that the two lancelet sequences are closely related to each other but clearly divergent from *Ciona intestinalis*, lamprey and tetrapod Nme1/Nme2 sequences. Similarly, the two *Ciona intestinalis *sequences are closely related to each other but highly divergent from other Nme1/Nme2 sequences. In this species, both genes are located on different chromosomes whereas in the lancelet they are located in tandem on the same chromosome. Altogether, these observations suggest that the Group I *Nme *gene pair arose from a cis-duplication of an ancestor gene in lancelet, whereas emergence of the two Group I *Nme *genes in ciona is more likely to be explained by a duplication followed by a translocation event. We thus hypothesize that in each species, the two genes result from an independent duplication event of an ancestor gene common to all chordates. This would be consistent with the number of Group I *Nme *genes in lamprey, as generation of *Nme2 *and *Nme3/4 *can be explained by the first round of whole genome duplication (1R) which occurred early in the vertebrate lineage [27]. The ancestor gene, from which emerged all Group I *Nme*, was thus named *NmeGroupI *(*NmeGp1*) (Fig. 8).

### Expression and putative functions of Nme proteins

#### Nme1/2-related proteins

**Figure 9:**
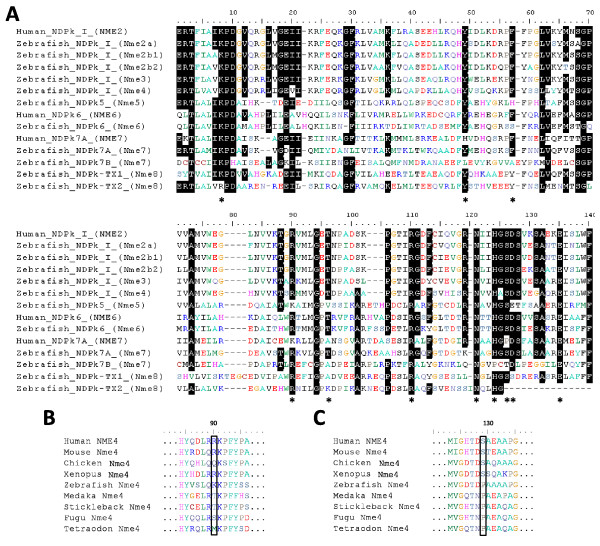
**Sequence alignment of zebrafish and human Nme protein domains and highlight on Nme4 specificity**. (A) Nme protein domains were identified using NCBI Conserved Domain Database [23]. Domains were aligned using MUSCLE [86], and graphic view was generated using BioEdit V. 7.0.9 software. Residues identified as important for kinase function catalytic mechanism are indicated by an * according to X-ray structure information on human NME2 [37] and Lascu and Gonin review [38] on the catalytic mechanism of NDP Kinase. (B) Mitochondrial membrane linkage triad with central Arginine^90^. (C) Proline to Serine mutation restricted to tetrapods. Sequences accession numbers are listed in Tables 1 and 2.

**Figure 10:**
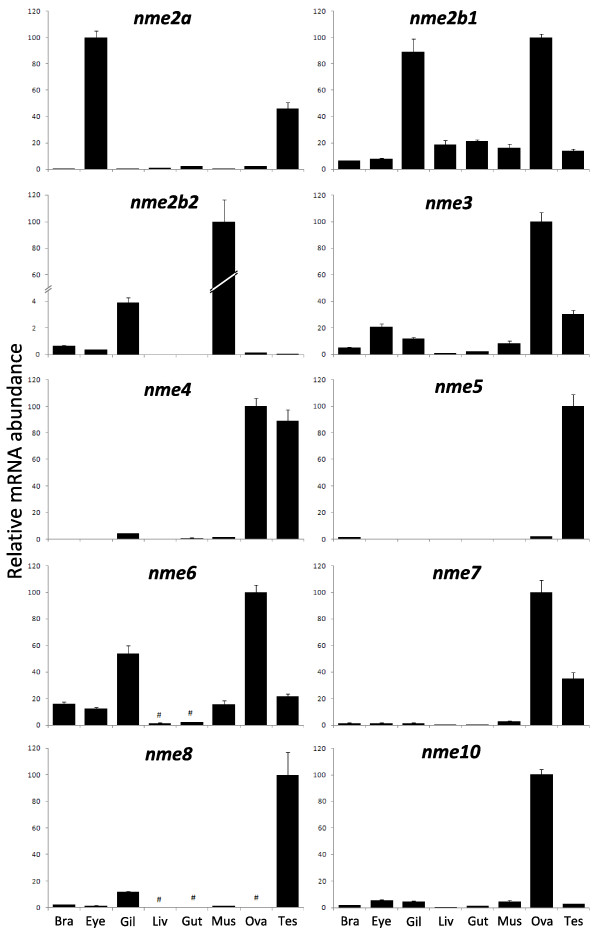
**Tissue distribution of zebrafish *nme *mRNAs**. Tissue expressions of zebrafish *nme2a*, *nme2b1*, *nme2b2*, *nme3*, *nme4*, *nme5*, *nme6*, *nme7*, *nme8*, and *nme10*. Real-time PCR analysis was conducted using total RNA originating from the following tissues sampled in three different sexually-mature females: brain (Bra), eyes (Eye), gills (Gil), hepatopancreas (Liv), intestine (Gut), muscle (Mus), ovary (Ova), and three different mature males: testis (Tes). For each tissue, three separate reverse transcription (RT) reactions were carried out using separate RNA samples originating from three different fish. RT reactions were pooled and use to run real-time PCR in quadruplicates. Mean and SD are shown (n = 4). #, Expression levels not significantly different from background signal at *p *< 0.05. For all genes, relative abundance is expressed in percentage of highest tissue expression after signal normalization by *18S *gene expression.

Given its role in metastatic dissemination, the Nme1 protein, has been extensively studied in humans and rodents [15,24]. A significant amount of data is also available for Nme2 [29]. Homologs of human genes were identified in several vertebrate species, such as rodents [6,30], cow [31], *Xenopus laevis *[20], zebrafish [19], salmon [18]; and non-vertebrate species such as scallop [32], drosophila [33], *Dictyostelium discoideum *[5], *Myxococcus Xanthus *[3], *Schizosaccharomyces pombe *[34] and various plants [35]. The orthology relationship of these Nme1/2-related proteins with human counterparts was not, however, always thoroughly characterized. Nme1/2-related proteins, as all Group I Nme, display a single NDPk_1 domain (Fig. 2), and various enzymatic assays demonstrated its kinase activity in different species [4,20,30-32,36]. According to our observations (Fig. 9A), the zebrafish Nme2 proteins display all the key residues for enzyme structure and activity [37,38] thus suggesting that Nme2 protein could exhibit a NDPK activity. Nme2 is widely expressed in adult tissues as shown in rat [39] and mouse [40,41]. During mouse embryogenesis, Nme2 protein accumulation is coincident with the functional differentiation of multiple organs [42]. No data are available about tissue expression of *Nme2 *in adult *Xenopus*. During *Xenopus laevis *early development, *Nme2 *transcripts cannot be detected before mid-blastula transition (MBT) but are expressed in differentiating tissues at later stages, thus suggesting an implication in cell differentiation and proliferation [17]. Our tissue distribution study has shown that the three *nme2 *zebrafish genes have very different tissue expression patterns (Fig. 10). In a previous study, an *nme2 *homolog was cloned in zebrafish [19]. This transcript, initially named *nme23-b*, corresponds to *nme2b1 *and was found to be expressed in hepatopancreas, head, ovary, and intestine by northern blot analysis. These observations are in total agreement with the broad tissues distribution of *nme2b1 *with a predominant expression in ovary and gills (Fig. 10) reported in the present study. In contrast to what is observed for *nme2b1*, zebrafish *nme2a *and *nme2b2 *have very specific tissue distributions (Fig. 10). It should however be stressed that, despite the extremely high expression in muscle, *nme2b2 *is also significantly expressed in all assayed tissues. Similarly *nme2a *expression is also weakly detected in all tissues in addition to the strong expression observed in eyes and testis. In Atlantic salmon, an *nme2*-related mRNA, belonging to the nme2a sub-family [See Additional file 6], is highly expressed in brain, and during early development it could not be detected before the end of gastrulation [18]. Altogether, the tissue distribution of the three zebrafish *nme2 *genes suggests that *nme2a *and *nme2b *genes have undergone specialization after duplication of a common ancestor *nme2 *gene [43]. Interestingly, Cañestro et al [44] recently demonstrated that in the case of the loss of one paralog after a duplication event, the surviving paralog can display combined expression pattern of both paralogs kept in another species. In the light of this conclusion, it would be interesting to study *nme2 *expression in species that lack the *nme2b *copy. Human NME2 was first identified as the PuF transcription factor that recognizes a nuclease hypersensitive site (NHE) motif in the *c-myc *promoter and stimulates transcription [29,45,46]. NME2 transcriptional activation of *c-myc *gene by binding to its promoter was confirmed in mouse [47] and *Xenopus laevis *[20]. Furthermore, Awd, the drosophila NME2 homolog, is required for proper differentiation and tissues morphology [12]. Thus, *NME2 *expression pattern during embryogenesis is consistent with implication in cell proliferation and differentiation. In addition, human NME2 may associate with estrogen receptor-β and is able to modulate estrogen-induced gene transcription [48]. Implication of NME2 in regulation of gene expression has also been demonstrated for other genes implicated in several biological processes including nuclease activity (for review see [49]). Altogether, available data suggest that vertebrates Nme2 proteins are involved in a wide variety of cellular processes that require further investigations.

#### Nme3

The Nme3 protein has been characterized in humans [50-52] and mice [53]. Nme3, as all the proteins of the Group I, displays a single NDPk_1 domain (Fig. 2). In humans, enzymatic activity could not be measured using the full length recombinant protein [36], but a truncated recombinant protein displayed kinase activity similar to that of the NME1 and NME 2 proteins [52]. We show here that zebrafish Nme3 possesses all the residues necessary for enzyme structure and activity [37,38] (Fig. 9A). Together, these observations would suggest an NDPk activity of the zebrafish Nme3. Zebrafish tissue distribution analysis showed that *nme3 *is expressed in all studied tissues with the strongest expression in the ovary, and a lower, but significant, expression in testis, eye and gills (Fig. 10). To our knowledge, the strong ovarian expression of *nme3 *has never been reported in vertebrates in a non-malignant context. In contrast, existing data indicate that human *NME3 *is ubiquitously expressed in non-metastatic tissues with a particularly strong expression in specific structures of the brain [15]. During mouse organogenesis, *Nme3 *is preferentially expressed in the nervous and sensory system [54], whereas in adult mouse, transcripts are found ubiquitously distributed with higher expression in brain and liver [53]. During *Xenopus laevis *embryogenesis, it was shown that *Nme3 *was predominantly expressed in the head region [55]. To date, very little is known about NME3 function in a non-malignant context. It was shown that over-expression of *NME3 *gene in 32Dc13 peripheral blood cells inhibited differentiation into granulocytes and caused apoptosis [50], without requiring NDPk enzymatic activity [56]. In addition, it was shown that NME3 induces morphological changes associated with neural differentiation in neuroblastoma cells [57] and that it could act on cell motility by enhancing the amount of integrin β [58]. In the *Xenopus laevis *it was shown that *Nme3 *was highly expressed in the ciliary marginal zone of the retina and involvement of Nme3 in cell fate determination during retinogenesis was therefore suggested [55]. It was also shown that *NME3 *was an estrogen-responsive gene in the context of mammary tumors [59]. To date, no information is available on the physiological or cellular functions of Nme3 in teleosts. However, an implication in cell differentiation, proliferation and apoptosis can be hypothesized.

#### Nme4

Nme4 protein has been characterized in humans [60], mouse [53], pigeon [61] and *Xenopus laevis *[55]. Nme4, as all Group I Nme, is composed of a single NDPk_1 domain (Fig. 2). Zebrafish Nme4 possesses all the residues necessary for enzyme structure and kinase activity [37,38] (Fig. 9A). In humans, the enzymatic activity of NME4 was experimentally confirmed [36,62]. As reported here (Fig. 9C), all studied Nme4 tetrapod proteins naturally display a serine residue at position 129, equivalent to the lethal *Killer of prune *(*K-pn*) mutation of the drosophila [12]. It was previously shown that the presence of Serine^129 ^residue has local structural effects that weaken subunit interactions and decreases hexamer stability [62]. Strikingly, teleost Nme4 sequences do not display the Serine^129^, but display the Proline^129 ^shared by all other Group I Nme members (Fig. 9C). The presence of this mutation in tetrapod proteins that cannot be found in any studied teleost species suggests that this mutation appeared just after the sarcopterigian radiation. It was recently shown that human NME4 binds the inner mitochondrial membrane and couples nucleotide transfer with respiration [63]. The binding property to mitochondrial membranes is due to electrostatic interactions between the central Arginine^90 ^of a triad of basic residue and anionic phospholipids [63]. A basic residue equivalent to Arg^90 ^can also be found in mouse, *Xenopus tropicalis *and zebrafish Nme4 (Fig. 9B). Tetraodon Nme4 possesses a hydrophobic methionine and might be able to electrostatically interact with anionic phospholipids too. In contrast, chicken and other studied teleost Nme4 sequences display a hydrophilic residue in position 90. This could suggest that these Nme4 are unable to interact with anionic phospholipids. It has been shown that pigeon Nme4, also displaying a hydrophilic 90-residue, is located in the mitochondrial matrix [61]. Many functions such as nucleotide supply, functional interactions with Krebs cycle succinyl thiokinase, catabolism of short chain fatty acids [64,65] and, more recently, GTP synthesis in relationship with iron homeostasis [66] have been proposed. In the present study, we report that zebrafish *nme4 *is highly and predominantly expressed in gonads, weakly expressed in gills, and barely detectable in other studied tissue (Fig. 10). In contrast, human *NME4 *was shown to be widely distributed and expressed in a tissue-dependant manner with a moderate expression in liver, muscle and ovary and a low expression in testis and brain [60]. In mouse, *Nme4 *was only detectable in heart, liver and kidney [53]. In *Xenopus laevis*, *Nme4 *is predominantly expressed in the head region and an indirect regulation of retinal gliogenesis by *Nme4 *was demonstrated [55]. The gonad-predominant expression of *nme4 *reported here, if confirmed in other teleost species, could suggest a different function of fish Nme4 in gonads in comparison to mammalian Nme4. However, a Relative Rate Test [67] did not reveal a significantly different evolutionary rate between tetrapods and fish (*p *= 0.70). This suggests that observed differences in expression patterns reported above are not linked to different evolutionary rates.

#### Nme5

Nme5 sequences have been characterized in humans [68] and mouse [69]. The zebrafish Nme5, as human NME5, is composed of an NDPk5 domain followed by a Dpy-30 domain (Fig. 2). In agreement with previous observations made in human and mouse [69], the zebrafish NDPk5 domain also lacks three of the eleven residues deemed crucial for enzyme structure and activity [37,38] (Fig. 9A). The lack of kinase activity was confirmed using human recombinant proteins [36,68]. However, a pronounced 3'→ 5' exonuclease activity was measured for human NME5 [36]. In zebrafish, *nme5 *was predominantly expressed in testis and detected at low levels in brain and ovary (Fig. 10). Our results are in total agreement with data obtained in humans [68] and mouse [69] in which a predominant testis expression was observed. Low expression levels were also detected in human brain and kidney [68] while a low expression of the mouse transcript was detected in ovary, heart, kidney, and brain [69]. In human testis, *NME5 *gene expression is located in spermatogonia and early spermatocytes [68], whereas expression appears at pachytene stages in mouse [69]. A marked delay in protein expression can be observed as Nme5 protein is only found in the flagella of spermatids and spermatozoa, adjacent to the central pair and outer doublets of axonemal microtubules [70]. Functionally, murine Nme5 protein might be involved in late spermiogenesis by increasing the ability of late-stage spermatids to eliminate reactive oxygen species [69,71]. Together, our observations suggest that, within Group II, the Nme5 protein of vertebrates probably lacks NDPK activity and might have evolved towards testicular functions, possibly in germ cells.

#### Nme6

To date, NME6 has only been sequenced and characterized in humans [72,73]. Zebrafish Nme6 displays a single NDPk6 domain, also found in the human protein [72,73] (Fig. 2). In contrast to human NME6, the zebrafish Nme6 lacks one of the eleven residues deemed crucial for enzyme structure and activity, i.e. Phenylalanine^58^, but display a Phe in position 59 [37,38](Fig. 9A)., Using *E. coli *recombinant proteins, it was shown that human NDPk6 domain exhibited a kinase activity [73]. This observation was, however, not confirmed in another study [36]. Zebrafish *nme6 *is expressed in all studied tissues apart from hepatopancreas and intestine, and the highest expression levels were observed in ovary and gills (Fig. 10). Our results are consistent with previous RT-PCR results showing that *NME6 *was expressed in every human tissue, with strongest expression in ovary/placenta, muscle and intestine [72,73]. Very little is known about NME6 function or expression in a non-malignant context. However, it has been hypothesized that NME6 protein was partially colocalized with mitochondria and that over expression in SAOS2 cells resulted in growth suppression and generation of multinucleated cells. Thus, NME6 may play a role in regulation of cell growth and cell cycle progression [73]. All together, our results suggest that zebrafish Nme6 could possess kinase activity and might have conserved a crucial role in cell cycle, growth or development.

#### Nme7

To date, very little is known about human NME7 [15]. The zebrafish Nme7, as human NME7, contains a DUF1126 domain, belonging to the DM10 family, and an NDPk_7A and an NDPk_7B domain (Fig. 2). Very little is known about the function of DUF1126 domain and its DM10 family. However, it was suggested that this domain family may act as flagellar NDPk regulatory modules or as units specifically involved in axonemal targeting or assembly [74]. In contrast to the human NDPk_7A domain, the zebrafish domain displays all the residues deemed crucial for enzyme structure and activity [37,38] (Fig. 9A). In addition, human and zebrafish NDPk_7B domain respectively lack 3 and 5 residues deemed crucial for enzyme structure and activity [37,38](Fig. 9A). Yoon *et al *[36] confirmed the lack of kinase activity in human NME7 but reported a marked exonuclease activity. Zebrafish *nme7 *is predominantly expressed in gonads and only a weak expression can be found in other studied tissue (Fig. 10). Our results are consistent with human *NME7 *expression which is predominantly expressed in testis and expressed at significant levels in ovary and brain [15].

#### Nme8 and Nme9

To date, Nme8 protein has only been described in humans and mice and was called SPTRX2 for its resemblance with another protein, SPTRX1, also displaying a thioredoxin domain [25,75]. An orthologous gene was also characterized in *Ciona intestinalis *[76]. Proteins of this family are made of one thioredoxin domain (TRX_NDPK) followed by three tandemly repeated NDP kinase domains (NDPk_TX) (Fig. 2). Nme8 protein domain structure is very well conserved between human and zebrafish, with the exception of the third zebrafish NDPk-TX domain which is truncated. NME9 protein was also only described in humans [77] and displays a thioredoxin domain associated to a NDPk_TX domain (Fig. 2). Despite their thioredoxin domain, no thioredoxin activity, corresponding to a general protein-disulfide reductase, could be detected neither in Nme8 [24] nor in Nme9 [77]. Our results also show that the zebrafish NDPk_TX domains lacks crucial amino acids for kinase activity [37,38] (Fig. 9A) and are consistent with several enzymatic studies [25,36,77]. Similarly to NME5 and 7, human NME8 exhibits exonuclease activity [36]. Zebrafish *nme8 *is highly and predominantly expressed in testis and significantly detected in gills in comparison to all other tissues (Fig. 10). This observation is in complete agreement with existing data in mammals [15,25,75]. As previously reported, NME8 protein have domain arrangement similarities with sea urchin IC1, a member of the dynein intermediate chain [25,76,78]. The functional implication of NME8 in sperm axonemal organization was suggested [75,76] and key role of NME8 in flagellar anomalies and primary ciliary dyskinesia was disclosed [79]. Human NME9 was also described as highly expressed in testis but also in lung and other ciliated cell containing tissue and able to associate with microtubules [77]. Together, these observations suggest that zebrafish Nme8 might also be implicated in testicular function, possibly in axonemal organization.

#### Nme10

Nme10, also called XRP2, is the most recently described member of the Nme family and was only characterized in human and mouse [80]. Vertebrate Nme10 proteins display a TBCC (Tubulin-specific chaperone protein co-factor C) domain and a partial NDPk domain (Fig. 2). The TBCC domain acts as a GTPase activating protein (GAP) for β-tubulin [24]. The zebrafish partial NDPk domain lacks many crucial amino acids for kinase activity, in particular the catalytic histidine [37,38]. The lack of NDPk activity in human NME10 was confirmed by enzymatic assay [80]. Similarly to NME5, 7 and 8, NME10 exhibits exonuclease activity [80]. Zebrafish *nme10 *is predominantly expressed in the ovary and only a weak expression can be found in other studied tissue (Fig. 10). In humans and mice, *Nme10 *was found to be expressed in a wide variety of tissues [81]. Strong ovarian expression was however never reported as no study used ovarian tissue to study *Nme10 *expression. In humans, mutation in the *NME10 *gene induce Retinitis Pigmentosa, the major form of heritable blindness [80]. Interestingly, the partial NDPk domain of NME10 protein may have important function as most disease-related mutations of the *NME10 *gene concern this part of the protein [81]. Furthermore, the human NME10 protein, shown to be mainly located into the cytoplasm, undergoes re-localization into the nucleus when cells are treated with DNA damaging agent inducing oxidative stress, thus suggesting a participation in DNA repairing reactions [80]. The roles of Nme10 in fish and all other non-mammalian species are currently unknown and deserve specific studies. The ovarian-predominant expression, if confirmed in other species, is rather intriguing as it could suggest a major role of Nme10 in oogenesis.

## Conclusion

In the present study, we provide a comprehensive overview of the evolutionary history of the Nme family in vertebrates (Fig. 8). We also provide a characterization of the *Nme *gene repertoire in several vertebrate species including non-mammalian species and propose a gene nomenclature that is consistent with existing mammalian nomenclature. Our observations show that vertebrate *Nme *genes can be separated in two evolutionary distinct groups. *Nme1*, *Nme2*, *Nme3*, and *Nme4 *belong to the Group I while vertebrate *Nme5*, *Nme6*, *Nme7*, *Nme8*, and *Nme9 *belong to the Group II. The position of *Nme10 *in the Group II is in contrast more debatable due to its very specific evolutionary history and the recent incorporation of an NDPk domain, before or around the gnathostome radiation. The present study clearly indicates that *Nme5*, *Nme6*, *Nme7*, and *Nme8 *originate from duplication events that occurred before the chordate radiation. Finally, we show that *Nme9 *is a mammalian-specific protein closely related to *Nme8 *that arose from the cis-duplication of the *Nme8/Nme9 *ancestor gene after the separation of eutherians and metatherians. In contrast to the Group II, *Nme *genes of the Group I have a totally different evolutionary history. Our observations suggest that a single Group I gene ancestor was present in the chordate ancestor genome. The first round of whole genome duplication (1R) then resulted in two distinct genes named *Nme2 *and *Nme3/4 *that can be found in the lamprey genome. In contrast, no duplicates seem to have been retained after the second round of whole genome duplication (2R). We provide evidence that the *Nme3/4 *gene was cis-duplicated, thus resulting in *Nme3 *and *Nme4 *genes that can be found in all investigated gnathostome genomes. Our analyses also suggest that the *Nme1 *gene found in mammals, chicken and lizard results from the duplication of the *Nme2 *gene that occurred after amphibian radiation. In teleosts, the third round of whole genome duplication (3R) resulted in the apparition of two paralogous genes, *nme2a *and *nme2b*. While *nme2a *could be found in all teleost genomes, *nme2b *underwent different fates depending on the species. Finally, based on protein structure and tissue expression of zebrafish *nme *genes, we provide new insights in tissue specificity and molecular functions of Nme proteins in vertebrates and raise intriguing questions on the role of Nme protein in the vertebrate gonads.

## Methods

### Sequence analysis

All Nme sequences were identified using the following genome assemblies: zebrafish (*Danio rerio*, Assembly ZV7), medaka (*Oryzias latipes*, Assembly MEDAKA1), stickleback (*Gasterosteus aculeatus*, Assembly BROAD S1), tetraodon (*Tetraodon nigroviridis*, Assembly V.7), fugu (*Takifugu rubripes*, Assembly V.4), Xenopus (*Xenopus tropicalis*, Assembly V.4.1), anole lizard (*Anolis carolinensis*, AnoCar1.0 Assembly), chicken (*Gallus gallus*, Assembly V.2.1), mouse (*Mus musculus*, Assembly NCBI m37), human (*Homo sapiens*, Assembly NCBI 36), lamprey (*Petromyzon marinus*, Preliminary assembly 5.9X), *Ciona intestinalis *(Assembly V.2.0) and lancelet (*Branchiostoma floridae*, Assembly V.2.0). A large number of sequences were obtained from NCBI NR database using human or zebrafish protein sequence as a query [82]. When more than one sequence was obtained, the RefSeq and/or the longest one were preferentially selected. When sequences were not available in NR database, BLASTP on Ensembl database [83], BLAT on UCSC Genome Bioinformatics [84,85] and TBLASTN on EST_OTHERS database on Genbank [82] were used. For cow (*Bos Taurus*, Assembly Btau_4.0), opossum (*Monodelphis domestica*, Assembly MonDom5) and platypus (*Ornithorhynchus anatinus*, Assembly Ornithorhynchus_anatinus-5.0) only sequences corresponding Nme1 and Nme2 proteins were searched for. In mammalian species, a read-through transcript over *Nme1 *and *Nme2 *genes, named *NmeLV*, was recently identified [26]. Protein sequences corresponding to this transcript were not kept in the phylogenetic reconstruction as they displayed in their sequence the complete Nme2 protein sequence, thus leading to uninformative additional information. However, sequences from human, chimpanzee (*Pan troglodytes*), horse (*Equus caballus*), cow, platypus and anole lizard were found as reported in Table 1. Chromosomal localization of *Nme *genes was performed using Ensembl genome browser, or with UCSC Genome Bioinformatics BLAT when not available on Ensembl. Sequences for each Nme family were aligned by using MUSCLE [86] with default multiple alignment parameters and identity matrix were obtained with BioEdit 7.0.9 software. Intron-exon structure was obtained through Ensembl database, or, when no information was available, by species genome assembly Blat of protein and RNA sequences to get coding and non-coding intron-exon structure. The protein domain structure of Nme proteins was compared between human and zebrafish using Genbank Conserved Domain Database [23]. Domains defined by GenBank Conserved Domain Database were extracted from total protein sequence and aligned using MUSCLE.

### Phylogenetic analyses of Nme proteins

Phylogenetic reconstructions were performed using the automated genomic annotation platform FIGENIX [87]. All protein sequences of the Nme family were added to a single multiple alignment to assess their phylogenetic relationships. Sequence alignment was performed automatically by FIGENIX pipeline using MUSCLE. Alignment of sequences of different length and repeated domains present some difficulties due to domains similarities. Therefore, concerning sequences displaying repeated domains, alignment was performed using the part of the sequence showing the highest homology with sequences displaying a single domain. The sequence alignment used for phylogenetic analysis of the whole family is given in Additional file 7. The pipeline used is based on three different methods of phylogenetic tree reconstruction, i.e. Neighbour Joining, Maximum Parsimony, and Maximum likelihood and a midpoint-rooted consensus tree was built. Bootstrapping was carried out with 1000 replications. Bootstrap values are reported for each method when a node exists as identical in the three trees. However, sometimes a node only exist in one or two methods, and therefore * indicates that this node does not exist in the corresponding tree. The Nme1-Nme2 subtree was removed from the main tree and studied separately between tetrapods and teleosts because of different evolutionary history and high similarities leading to non-usable phylogenetic reconstruction.

### Relative Rate Test

For Nme4, a higher evolutionary rate between tetrapods and teleost was hypothesized according to major differences in expression patterns. A Relative Rate Test was therefore performed using the *Plasmodium falciparum *Nme protein [GenBank: XP_001350376] as an outgroup and using the RRTree software [67]. Input alignment file was generated using MUSCLE. RRTree is a user-friendly program for comparing substitution rates between lineages of protein or DNA sequences, relative to an outgroup. Genetic diversity is taken into account through the use of sequences from several species.

### Synteny analysis

The synteny relationships of *Nme1 *and *Nme2 *members over tetrapods genomes were analyzed using CASSIOPE (Clever Agent System for Synteny Inheritance and Other Phenomena in Evolution) [88]. Briefly, CASSIOPE integrates two important steps in a single automated process: (1) the phylogeny: orthologous/paralogous genes are determined by the aggregation of three phylogenetic methods using the Figenix plateform [87]. Additionally, phylogenetic information allows reconstruction of the evolutionary history and thereby a more accurate ancestral genome reconstruction (2) a statistical test: CASSIOPE therefore utilizes a specific statistical test to assess the significance of the predicted, conserved gene clusters on chromosomes. CASSIOPE does not perform synteny analysis on Scaffolds. As most teleost *nme2 *genes are located on Scaffolds, synteny analyses of *nme2a *and *nme2b *members in fish was thus conducted manually using Ensembl database putative orthology relationships [89].

### Zebrafish tissues sampling

Investigations were conducted according to the international guiding principles for the use and care of laboratory animals and in compliance with French and European regulations on animal welfare (DDSV approval #35-31). Three mature female zebrafish were obtained from the fish rearing facilities at INRA-SCRIBE (Rennes, France), over anesthetized and tissues immediately sampled, snap-frozen in liquid nitrogen and conserved at -80°C until RNA extraction. Testis samples were also obtained from three different males.

### Real-Time PCR analyses

For each tissue sample, total RNA was isolated using Tri-Reagent^® ^(Molecular Research Center, Cincinnati, OH) according to the manufacturer's instructions. Reverse transcription (RT) was performed as previously described [90] using 2 μg of RNA for each sample with M-MLV enzyme and Random Primers (Promega, Madison, WI). For each studied tissue, cDNA originating from three individual fish were pooled and subsequently used for real-time PCR. Control reactions were run without reverse transcriptase and used as negative control in the real-time PCR study. Quantitative RT-PCR experiments were performed using an Applied Biosystems StepOnePlus. RT products, including control reactions, were diluted to 1/25, and 4 μl was used for each real-time PCR. All q-RT-PCR reactions were performed in quadruplicates. Real-time PCR was performed using a real-time PCR kit provided with a Fast-SYBR^® ^Green fluorophore (Applied Biosystems) with either 200 or 300 nM of each primer. In order to avoid genomic DNA contamination bias, primers were designed on exon junctions. Primer sequences are listed in Additional file 8. The relative abundance of target cDNA within a sample set was calculated from serially diluted cDNA pool (standard curve) using Applied Biosystem StepOne™ V.2.0 software. After amplification, a fusion curve was obtained to validate the amplification of a single PCR product. The fusion curves obtained showed that each primer pair used was specific of a single *nme *transcript. Normalization of gene expression by 18*S *and *ef1a *resulted in similar results. Before further analysis, real-time PCR data were normalized using 18S transcript abundance in samples diluted to 1/2000 and with 100 nM of each primer. The control reactions were used to calculate background expression level for each gene to identify tissues exhibiting expression levels significantly higher than background.

## Authors' contributions

TD performed the experiments, produced the figures and drafted the manuscript. PP participated to the phylogenetic reconstruction and in the writing of the manuscript. JB participated in experiments and data analysis. CF and JB conceived and coordinated the study and participated in the writing of the manuscript. All authors read and approved the final manuscript.

## Supplementary Material

Additional file 1 **Identity matrices for Nme8 and Nme10 among chordates**. For Nme8 and Nme10, each protein was compared to all cognate chordates proteins. Multiple alignments were performed with MUSCLE and identity matrices generated by BioEdit 7.0.9 software. Click here for file

Additional file 2 **Identity matrices for Nme3 to Nme5 among chordates**. For Nme3 and Nme4, each protein was compared to all cognate vertebrate proteins, and to all cognate chordate proteins for Nme5. Multiple alignments were performed with MUSCLE and identity matrices generated by BioEdit 7.0.9 software. Click here for file

Additional file 3 **Identity matrices for Nme6 and Nme7 among chordates**. For Nme6 and Nme7, each protein was compared to all cognate chordates proteins. Multiple alignments were performed with MUSCLE and identity matrices generated by BioEdit 7.0.9 software. Click here for file

Additional file 4 **Phylogenetic reconstruction of the Nme protein family in teleosts**. Phylogenetic tree was constructed from a single multiple alignment. Bootstrap values for neighbour joining, maximum parsimony, and maximum likelihood methods, respectively, are indicated for each node. * indicates that the node does not exist in the corresponding tree. The consensus tree was calculated using the FIGENIX [87] automated phylogenomic annotation pipeline. Nme1-Nme2 subtree was removed from the main tree and studied separately. For each sequence, NCBI or Ensembl accession number and species name are shown. Click here for file

Additional file 5 **Identity matrices for Nme1 and Nme2 among vertebrates**. Fish Nme2, tetrapods Nme1 and tetrapods Nme2 were studied separately. Multiple alignments were performed with MUSCLE and identity matrices generated by BioEdit 7.0.9 software. Click here for file

Additional file 6 **Phylogenetic reconstruction of Nme2 proteins in teleosts**. Teleost Nme2 phylogenetic trees were constructed from separate multiple alignments. Bootstrap values for neighbor joining, maximum parsimony, and maximum likelihood methods, respectively, are indicated for each node. * indicates that the node does not exist in the corresponding tree. The consensus tree was calculated with the FIGENIX automated phylogenomic annotation pipeline [87]. For each sequence, accession number and species name are shown. Click here for file

Additional file 7 **Alignment of chordate Nme proteins**. Sequence alignment generated and used by FIGENIX for chordate Nme protein phylogenetic reconstruction. Click here for file

Additional file 8 **Primer used for the real-time PCR study**. For each target gene, abbreviated names, GenBank accession number of the corresponding zebrafish sequence and primer sequences are shown. Click here for file
