# Supplementary material for: Nme protein family evolutionary history, a vertebrate perspective
Source: BMC Evol Biol. 2009 Oct 23;9:256. doi: 10.1186/1471-2148-9-256 (PMC2777172; doi:10.1186/1471-2148-9-256)
Supplement: Additional file 1 — Identity matrices for Nme8 and Nme10 among chordates. For Nme8 and Nme10, each protein was compared to all cognate chordates proteins. Multiple alignments were performed with MUSCLE and identity matrices generated by BioEdit 7.0.9 software. [file 1471-2148-9-256-S1.PDF]

Nme8

	B. floridae	*C. intestinalis*	*H. sapiens*	*M. musculus*	*G. gallus*	*A. carolinensis*	*X. tropicalis*	*D. rerio*	*O. latipes*	*G. aculeatus*	*T. rubripes*	*T. nigroviridis*
Branchiostoma floridae **(XP_002217610)**	100	45.7	34.9	33.2	43.6	41.3	47.4	43.1	41.9	48.6	48.4	48.7
Ciona intestinalis **(NP_001027618)**		100	30.3	27.8	36.9	35.9	46.2	38.9	36.2	41.5	42.3	41.9
Homo sapiens **(NP_057700)**			100	62.5	45.5	45.4	36.1	31.9	37.7	38.7	40.5	40.8
Mus musculus **(NP_853622)**				100	43.5	42.2	35	31.8	36.8	37.3	37.7	36.9
Gallus gallus **(XP_426021)**					100	56.6	48.3	41.2	41.4	46.6	46.2	45.3
Anolis carolinensis **(ENSACAP00000017404)**						100	45.4	39.9	42.4	46.3	47.1	47.1
Xenopus tropicalis **(NP_001121456)**							100	54	52.5	58.6	55.3	53.8
Danio rerio **(NP_001082944)**								100	51.7	61	56.3	56
Oryzias latipes **(ENSORLP00000020619)**									100	65.1	62.4	60.1
Gasterosteus aculeatus **(ENSGACP00000008024)**										100	72.3	69.8
Takifugu rubripes **(ENSTRUP00000026461)**											100	81.9
Tetraodon nigroviridis **(ENSTNIP00000019567)**												100

Nme10

	B. floridae	*C. intestinalis*	*H. sapiens*	*M. musculus*	*G. gallus*	*A. carolinensis*	*X. tropicalis*	*D. rerio*	*O. latipes*	*G. aculeatus*	*T. rubripes*	*T. nigroviridis*
Branchiostoma floridae **(XP_002243612)**	100	41.1	56.6	54.8	58.2	58.2	54.9	52.4	53.3	51.2	54.4	53.3
Ciona intestinalis **(ENSCINP00000026020)**		100	38.4	38.2	36	36.8	36.6	34.5	37.4	37	37.2	36.6
Homo sapiens **(NP_008846)**			100	87.7	78.4	72.5	71.7	63.2	62.8	64	63.8	64.5
Mus musculus **(NP_598430)**				100	75	71.1	69.7	62.9	60.9	62.9	63	62.9
Gallus gallus **(NP_001008680)**					100	76.1	75	63.9	64.4	64.8	64.4	64.7
Anolis carolinensis **(ENSACAP00000011842)**						100	71.9	68.1	62.2	62.4	63.3	63.1
Xenopus tropicalis **(ENSXETP00000008933)**							100	62.2	66.9	65.1	68.2	67.4
Danio rerio **(NP_998611)**								100	73.2	70	73	71.5
Oryzias latipes **(ENSORLP00000020082)**									100	78.1	86.9	84.1
Gasterosteus aculeatus **(ENSGACP00000018275)**										100	80	78.2
Takifugu rubripes **(ENSTRUP00000027655)**											100	93
Tetraodon nigroviridis **(CAG01390)**												100
