# Supplementary material for: Nme protein family evolutionary history, a vertebrate perspective
Source: BMC Evol Biol. 2009 Oct 23;9:256. doi: 10.1186/1471-2148-9-256 (PMC2777172; doi:10.1186/1471-2148-9-256)
Supplement: Additional file 2 — Identity matrices for Nme3 to Nme5 among chordates. For Nme3 and Nme4, each protein was compared to all cognate vertebrate proteins, and to all cognate chordate proteins for Nme5. Multiple alignments were performed with MUSCLE and identity matrices generated by BioEdit 7.0.9 software. [file 1471-2148-9-256-S2.PDF]

Nme3

	H. sapiens	*M. musculus*	*G. gallus*	*A. carolinensis*	*X. tropicalis*	*D. rerio*	*O. latipes*	*G. aculeatus*	*T. rubripes*	*T. nigroviridis*
Homo sapiens **(NP_002504)**	100	88.1	75.1	66.2	72.1	72.7	69.2	66.2	66	68.6
Mus musculus **(NP_062704)**		100	73.9	66.8	70.4	71.5	69.8	66.8	64.3	67.4
Gallus gallus **(XP_414714)**			100	69.8	73.9	69.2	72.7	68	70.1	71
Anolis carolinensis **(ENSACAP00000003455)**				100	66.8	64.4	66.2	63.9	58.4	61.5
Xenopus tropicalis **(NP_001005115)**					100	72.7	70.4	65.6	67.2	69.2
Danio rerio **(NP_571003)**						100	75.1	72.7	72.5	70.4
Oryzias latipes **(ENSORLP00000014699)**							100	79.8	76.4	76.7
Gasterosteus aculeatus **(ENSGACP00000017241)**								100	74.2	75.1
Takifugu rubripes **(ENSTRUP00000011464)**									100	84.1
Tetraodon nigroviridis **(CAG02649)**										100

Nme4

	H. sapiens	*M. musculus*	*G. gallus*	*A. carolinensis*	*X. tropicalis*	*D. rerio*	*O. latipes*	*G. aculeatus*	*T. rubripes*	*T. nigroviridis*
Homo sapiens **(NP_005000)**	100	81.4	54.7	43.3	63.5	59.7	50	53.6	49.2	47.7
Mus musculus **(NP_062705)**		100	51.8	43.5	60.6	59.7	48	52	48.2	45.7
Gallus gallus **(AAB99857)**			100	46.8	52.1	52.6	45.2	54	45	42
Anolis carolinensis **(ENSACAP00000016602)**				100	44	44.2	40.2	45.6	43.3	41.2
Xenopus tropicalis **(NP_001039239)**					100	61.5	50.7	54.5	48.5	48
Danio rerio **(NP_957489)**						100	49.7	54.2	48.5	48.5
Oryzias latipes **(ENSORLP00000014089)**							100	62	54.6	56.6
Gasterosteus aculeatus **(ENSGACP00000016999)**								100	60.8	58.2
Takifugu rubripes **(ENSTRUP00000012573)**									100	85.1
Tetraodon nigroviridis **(CAG12673)**										100

Nme5

	B. floridae	*C. intestinalis*	*P. marinus*	*H. sapiens*	*M. musculus*	*G. gallus*	*A. carolinensis*	*X. tropicalis*	*D. rerio*	*O. latipes*	*G. aculeatus*	*T. rubripes*	*T. nigroviridis*
Branchiostoma floridae **(XP_002211295)**	100	57.3	56.8	68.3	67.7	64.6	67.2	66.3	64.5	53.8	56.4	54.6	55.4
Ciona intestinalis **(XP_002127400)**		100	43.4	49.8	48.8	44.7	49.2	48.2	48.2	40.3	42.9	40.9	38.7
Petromyzon marinus **(DW023083)**			100	52.8	52.6	51.8	52.1	54.2	53.9	48.7	44.9	50.7	48.2
Homo sapiens **(NP_003542)**				100	85.8	73.1	78.7	71.9	62.6	54.7	56.4	57.9	54.2
Mus musculus **(NP_542368)**					100	71.6	75.8	69.6	62.6	52.6	56.9	59.3	58.2
Gallus gallus **(XP_414687)**						100	74	67.2	61.7	52.3	54.6	55.6	54.7
Anolis carolinensis **(ENSACAP00000016699)**							100	68.6	64.9	55.4	58.3	57.4	57.3
Xenopus tropicalis **(NP_001072619)**								100	60.7	55.1	60	60.7	57.9
Danio rerio **(NP_001002516)**									100	54.8	62.8	57.9	55.7
Oryzias latipes **(ENSORLP00000006672)**										100	57.4	57.4	58.2
Gasterosteus aculeatus **(ENSGACP00000023932)**											100	61.4	60.6
Takifugu rubripes **(ENSTRUP00000001405)**												100	76.9
Tetraodon nigroviridis **(CAG01205)**													100
