# Supplementary material for: Nme protein family evolutionary history, a vertebrate perspective
Source: BMC Evol Biol. 2009 Oct 23;9:256. doi: 10.1186/1471-2148-9-256 (PMC2777172; doi:10.1186/1471-2148-9-256)
Supplement: Additional file 3 — Identity matrices for Nme6 and Nme7 among chordates. For Nme6 and Nme7, each protein was compared to all cognate chordates proteins. Multiple alignments were performed with MUSCLE and identity matrices generated by BioEdit 7.0.9 software. [file 1471-2148-9-256-S3.PDF]

Nme6

	B. floridae	*C. intestinalis*	*P. marinus*	*H. sapiens*	*M. musculus*	*G. gallus*	*A. carolinensis*	*X. tropicalis*	*D. rerio*	*O. latipes*	*G. aculeatus*	*T. rubripes*	*T. nigroviridis*
Branchiostoma floridae (XP_002217997)	100	34.2	49	44.8	45.5	47.3	49.4	46.3	48.2	51.1	44	53.2	52.2
Ciona intestinalis (ENSCINP00000027945)		100	38.2	35	37	34.4	34.6	36.3	38	39.7	32.1	39.1	38.6
Petromyzon marinus (EE741045)			100	50	50.7	53.2	58.1	53.6	53.4	57.3	52.1	60.3	57.9
Homo sapiens (NP_005784)				100	84.2	63.4	57.2	61.8	57.2	55.6	45.8	56.1	57.7
Mus musculus (NP_061227)					100	64	55.7	61.9	57.6	56.6	46.5	56	57.1
Gallus gallus (XP_424474)						100	60.3	59.6	55.9	57.5	43.5	57.5	58.6
Anolis carolinensis (ENSACAP00000008165)							100	55.7	58.4	56.8	52.1	62.7	60.6
Xenopus tropicalis (NP_001123709)								100	63.6	62.5	46.9	61.1	62.5
Danio rerio (NP_571672)									100	67.6	50.2	67.2	70.4
Oryzias latipes (ENSORLP00000020366)										100	58.6	77.2	80.6
Gasterosteus aculeatus (ENSGACP00000014120)											100	64.4	59.7
Takifugu rubripes (ENSTRUP00000019600)												100	82.2
Tetraodon nigroviridis (ENSTNIP00000019439)													100

Nme7

	B. floridae	*C. intestinalis*	*H. sapiens*	*M. musculus*	*G. gallus*	*A. carolinensis*	*X. tropicalis*	*D. rerio*	*O. latipes*	*G. aculeatus*	*T. rubripes*	*T. nigroviridis*
Branchiostoma floridae (XP_002244666)	100	51.1	54	51.5	52.4	45.6	54.8	51.4	37.3	50.1	49.8	50.9
Ciona intestinalis (XP_002128907)		100	52.1	52.1	54.9	48.8	54.4	53.7	39.4	53.9	52.6	50.8
Homo sapiens (NP_037462)			100	83.7	76.8	64	67.5	64	47.8	62.2	60.1	60.1
Mus musculus (NP_612187)				100	70.8	61.2	63	60.7	44.5	59.2	58.4	57.9
Gallus gallus (ENSGALP00000024531)					100	70.3	67.8	63	46.8	61.1	58.7	57.4
Anolis carolinensis (ENSACAP00000010447)						100	61.3	57.6	42.3	55.8	54.4	54.4
Xenopus tropicalis (NP_988903)							100	66.2	50.7	65.1	63.2	62.5
Danio rerio (NP_571004)								100	55	74.5	70.4	68.7
Oryzias latipes (DK039970)									100	58.8	56.9	56.1
Gasterosteus aculeatus (ENSGACP00000018067)										100	73.6	72.9
Takifugu rubripes (ENSTRUP00000012937)											100	83.2
Tetraodon nigroviridis (ENSTNIP00000004782)												100
