# Supplementary figures and images for: Nme protein family evolutionary history, a vertebrate perspective
Source: BMC Evol Biol. 2009 Oct 23;9:256. doi: 10.1186/1471-2148-9-256 (PMC2777172; doi:10.1186/1471-2148-9-256)

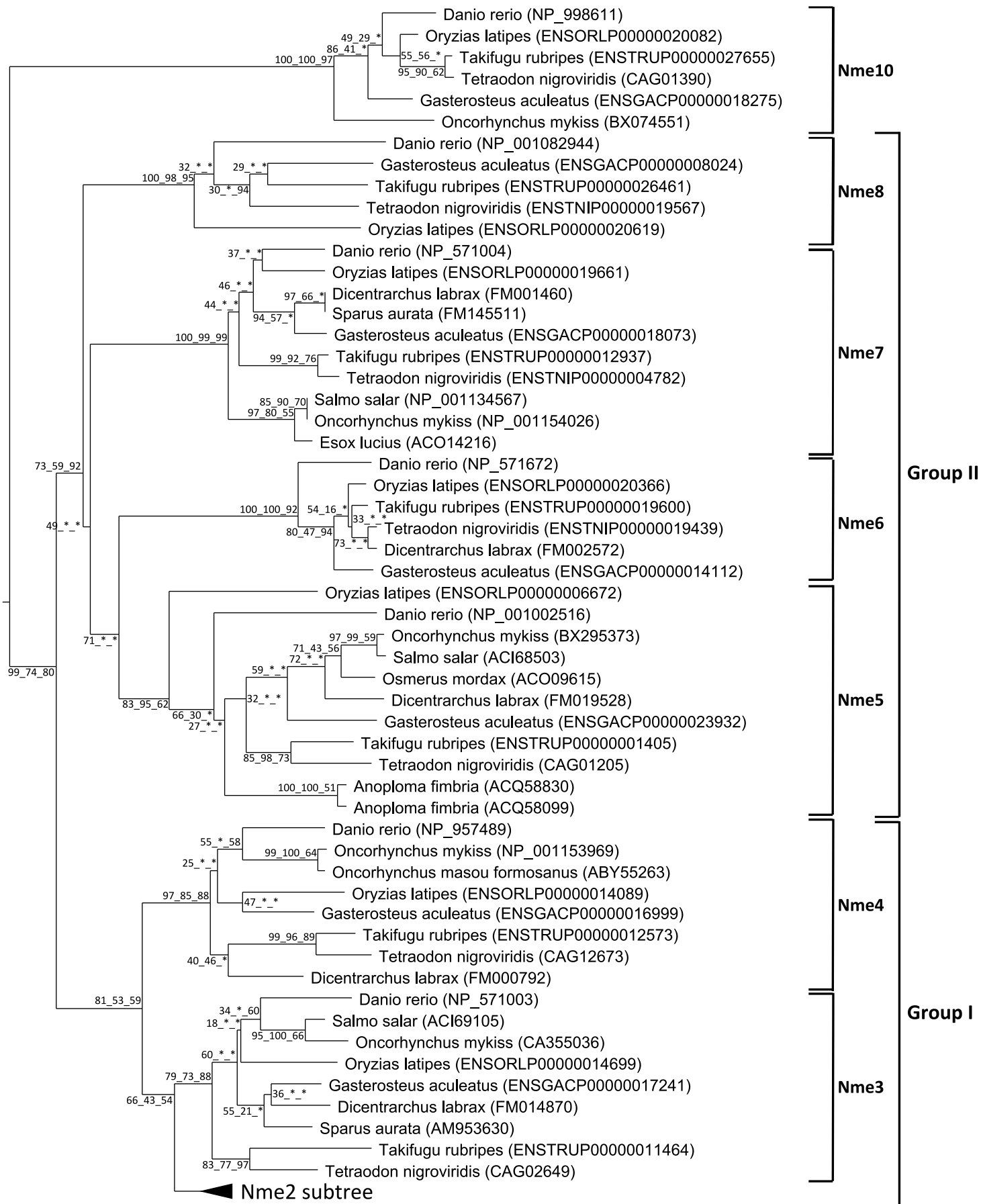

Supplement: Additional file 4 — Phylogenetic reconstruction of the Nme protein family in teleosts. Phylogenetic tree was constructed from a single multiple alignment. Bootstrap values for neighbour joining, maximum parsimony, and maximum likelihood methods, respectively, are indicated for each node. * indicates that the node does not exist in the corresponding tree. The consensus tree was calculated using the FIGENIX [87] automated phylogenomic annotation pipeline. Nme1-Nme2 subtree was removed from the main tree and studied separately. For each sequence, NCBI or Ensembl accession number and species name are shown. [file 1471-2148-9-256-S4.PDF]

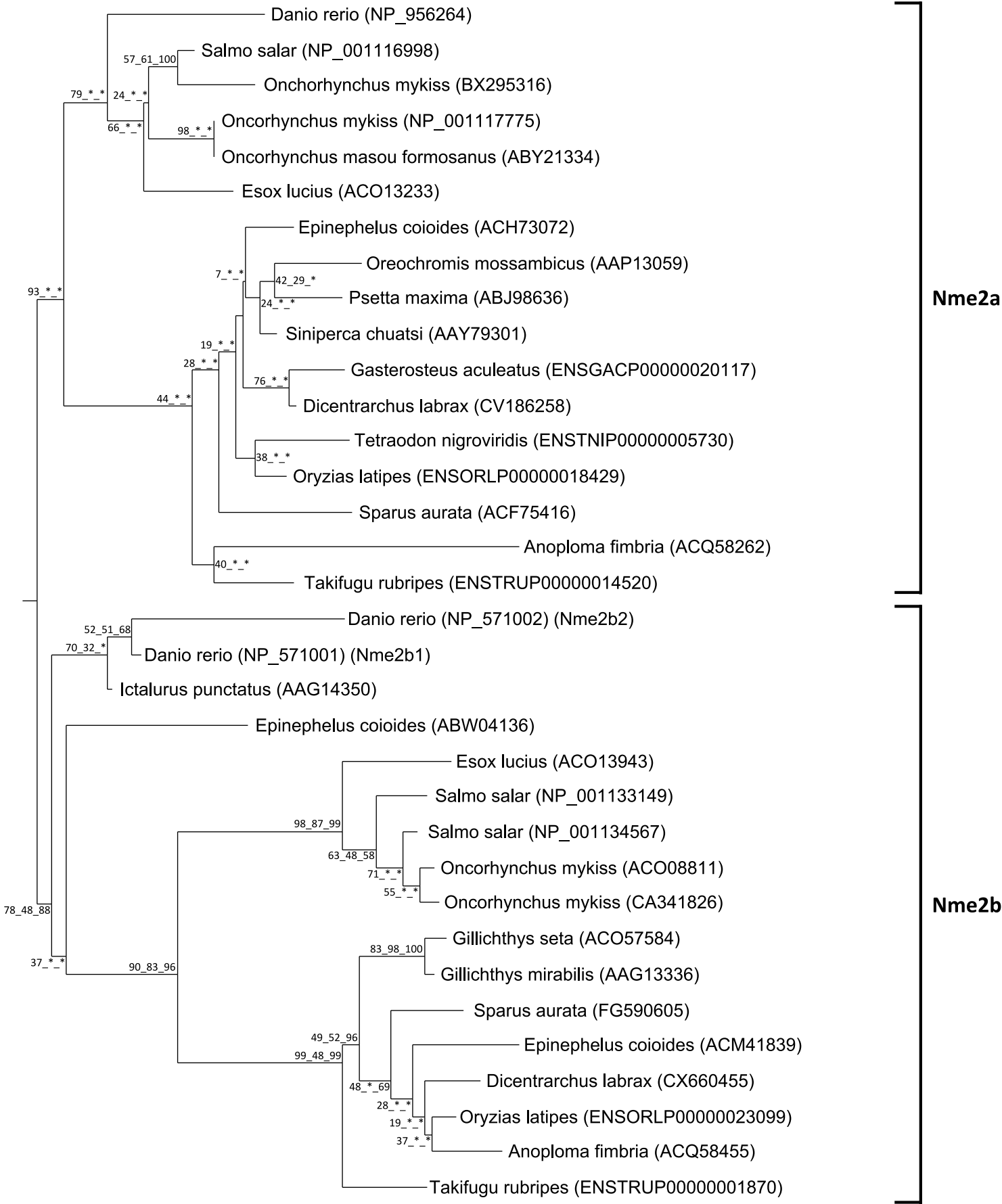

Supplement: Additional file 6 — Phylogenetic reconstruction of Nme2 proteins in teleosts. Teleost Nme2 phylogenetic trees were constructed from separate multiple alignments. Bootstrap values for neighbor joining, maximum parsimony, and maximum likelihood methods, respectively, are indicated for each node. * indicates that the node does not exist in the corresponding tree. The consensus tree was calculated with the FIGENIX automated phylogenomic annotation pipeline [87]. For each sequence, accession number and species name are shown. [file 1471-2148-9-256-S6.PDF]
