# Supplementary material for: Nme protein family evolutionary history, a vertebrate perspective
Source: BMC Evol Biol. 2009 Oct 23;9:256. doi: 10.1186/1471-2148-9-256 (PMC2777172; doi:10.1186/1471-2148-9-256)
Supplement: Additional file 5 — Identity matrices for Nme1 and Nme2 among vertebrates. Fish Nme2, tetrapods Nme1 and tetrapods Nme2 were studied separately. Multiple alignments were performed with MUSCLE and identity matrices generated by BioEdit 7.0.9 software. [file 1471-2148-9-256-S5.PDF]

Nme1

	H. sapiens	*M. musculus*	*B. taurus*	*M. domestica*	*O. anatinus*	*G. gallus*	*A. carolinensis*
***Homo sapiens* (NP_937818)**	100	80.7	80.2	76.8	78.5	70.6	74
***Mus musculus* (NP_032730)**		100	92.1	86.8	88.8	80.3	83.5
***Bos taurus* (NP_991387)**			100	88.8	88.8	82.3	86.8
***Monodelphis domestica* (XP_001363771)**				100	85.6	79.7	83.5
***Ornithorhyncus anatinus* (ENSOANP00000018628)**					100	83.6	86.2
***Gallus gallus* (XP_420097)**						100	86.9
***Anolis carolinensis* (ENSACAESTP00000008767)**							100

Nme2

	H. sapiens	*M. musculus*	*B. taurus*	*M. domestica*	*O. anatinus*	*G. gallus*	*A. carolinensis*	X. tropicalis
***Homo sapiens* (NP_001018149)**	100	98	93.4	95.3	92.8	91.5	86.9	84.4
***Mus musculus* (NP_032731)**		100	93.4	93.4	92.2	92.1	86.9	84.4
***Bos taurus* (NP_001069844)**			100	91.4	90.2	88.8	85.6	83.7
***Monodelphis domestica* (XP_001363684)**				100	91.5	87.5	84.9	81.8
***Ornithorhyncus anatinus* (ENSOANP00000018629)**					100	86.3	83.7	80
***Gallus gallus* (XP_990378)**						100	89.5	87.6
***Anolis carolinensis* (ENSACAESTP00000008779)**							100	81.1
***Xenopus tropicalis* (NP_001005140)**								100

Nme2a and Nme2b

	D. rerio Nme2a	*G. aculeatus* Nme2a	*O. latipes* Nme2a	*T. nigroviridis* Nme2a	*T. rubripes* Nme2a	*D. rerio* Nme2b1	*D. rerio* Nme2b2	*O. latipes* Nme2b	*T. rubripes* Nme2b
**Danio rerio (NP_956264) Nme2a**	100	72.5	76.4	73.8	64.6	77.7	71.2	50.9	62.7
***Gasterosteus aculeatus* (ENSGACP00000020117) Nme2a**		100	89.4	84.2	75.9	75.8	68.6	48	63.3
***Oryzias latipes* (ENSORLP00000018429) Nme2a**			100	88.1	79.5	80.3	71.8	49.3	66
***Tetraodon nigroviridis* (ENSTNIP00000005730) Nme2a**				100	79.5	77.1	71.2	49.3	66
***Takifugu rubripes* (ENSTRUP00000014520) Nme2a**					100	70.6	64	45.7	60.4
***Danio rerio* (NP_571001) Nme2b1**						100	85.6	57.5	73.2
***Danio rerio* (NP_571002) Nme2b2**							100	54.2	70.5
***Oryzias latipes* (ENSORLP00000023099) Nme2b**								100	65.7
***Takifugu rubripes* (ENSTRUP00000001870) Nme2b**									100
