# Supplementary material for: Nme protein family evolutionary history, a vertebrate perspective
Source: BMC Evol Biol. 2009 Oct 23;9:256. doi: 10.1186/1471-2148-9-256 (PMC2777172; doi:10.1186/1471-2148-9-256)
Supplement: Additional file 8 — Primer used for the real-time PCR study. For each target gene, abbreviated names, GenBank accession number of the corresponding zebrafish sequence and primer sequences are shown. [file 1471-2148-9-256-S8.PDF]

Target gene	GenBank Acc#	Forward sequence	Reverse sequence
18S	XM_001922869	CGGAGGTTCGAAGACGATCA	GAGGTTTCCCGTGTTGAGTC
ef1a	NM_131263	AAGGACATCCGTCGTGGTAA	GATGATGACCTGAGCGTTGA
nme2A	NM_199970	GACTGCCATCAAACAATTCCAA	AAGATCCTCATCTGCCTGGATTA
nme2B1	NM_130926	CCAGACGGGTTCTGTTCTGT	TGCTTGAGAAAAGACGAGATGA
nme2B2	NM_130927	TGGAGCTCAGAGTCCCTGTT	GGGTTCTGCTGTGTGTGTGT
nme3	NM_130928	TCCTGCACGGAGAAGATGAT	AACTCCATCCGGCTTCACT
nme4	NM_201195	TCAGCTGTTTGTGTGGCATT	TAACCCTCGGACCGTTACAC
nme5	NM_001002516	GACGCATCCTGACTGTCTGA	ATGGGGAAACATGAACCTGA
nme6	NM_131597	GCACACATGAGGATCACTGG	CATCAAGTGCAGCAGAAGGA
nme7	NM_130929	GGTTGTTGCCGAGTACCCTA	CATCTGGGGCATGTATTTCC
nme8	NM_001089475	ATCAAGAACGAGCTGGGAGA	TCCCGCATAAAACAGAAAGG
nme10	NM_213446	AATGGATCACCAAAGGTCCA	GCTTGGTTCCGTTGAACACT
